# Early development of the cochlea of the common marmoset, a non-human primate model

**DOI:** 10.1186/s13064-022-00162-8

**Published:** 2022-05-07

**Authors:** Makoto Hosoya, Masato Fujioka, Junko Okahara, Sho Yoshimatsu, Hideyuki Okano, Hiroyuki Ozawa

**Affiliations:** 1 Department of Otorhinolaryngology, Head and Neck Surgery, Keio University School of Medicine, 35 Shinanomachi Shinjuku-ku, Tokyo, 160-8582 Japan; 2 Present Address: Department of Molecular Genetics, Kitasato University School of Medicine, 1-15-1 Kitasato, Minami-ku, Sagamihara, Kanagawa, 252-0374 Japan; 3 Laboratory for Marmoset Neural Architecture, Center for Brain Science, RIKEN, 2-1 Hirosawa Wako, Saitama, 351-0193 Japan; 4 Department of Marmoset Biology and Medicine, Central Institute for Experimental Animals, 3-25-12 Tonomachi Kawasaki-ku Kawasaki, Kanagawa, 210-0821 Japan; 5 Department of Physiology, Keio University School of Medicine, 35 Shinanomachi Shinjuku-ku, Tokyo, 160-8582 Japan

**Keywords:** Cochlea, Marmoset, Rodents, Sensory epithelium, Cochlear development

## Abstract

**Background:**

Fine-tuned cochlear development is essential for hearing. Owing to the difficulty in using early human fetal samples, most of our knowledge regarding cochlear development has been obtained from rodents. However, several inter-species differences in cochlear development between rodents and humans have been reported. To bridge these differences, we investigated early otic development of a non-human primate model animal, the common marmoset (*Callithrix jacchus*).

**Methods:**

We examined 20 genes involved in early cochlear development and described the critical developmental steps for morphogenesis, which have been reported to vary between rodents and marmosets.

**Results:**

The results revealed that several critical genes involved in prosensory epithelium specifications showed higher inter-species differences, suggesting that the molecular process for hair cell lineage acquisition in primates differs considerably from that of rodents. We also observed that the tempo of cochlear development was three times slower in the primate than in rodents.

**Conclusions:**

Our data provide new insights into early cochlear development in primates and humans and imply that the procedures used for manipulating rodent cochlear sensory cells cannot be directly used for the research of primate cells due to the intrinsic inter-species differences in the cell fate determination program.

## Background

Auditory perception is the process by which mechanical sound waves are detected by the inner ear and converted into neuronal electrical impulses perceived by the brain. A coiled organ, the cochlea, plays an essential role in hearing during this sequential process in the inner ear. In the cochlea, hair cells convert mechanosensory sound waves into neural electrical pulses, which are transmitted by the synapses between the hair cells and spiral ganglion neurons and eventually reach the brain's auditory cortex [1, 2].

Fine-tuned cochlear development is essential for the acquisition of hearing. This development requires several well-controlled steps, including the formation of coiled structure, specification of sensory epithelium, differentiation of hair cells and spiral ganglion neurons, and their subsequent maturation  [3, 4]. Most of our knowledge regarding cochlear development has been obtained from rodent models, specifically from mice and rats. However, several inter-species differences in cochlear development between rodents and humans have been reported. For example, the expression pattern of PRPH (peripherin), a neurofilament of the spiral ganglion neurons, differs between primates and rodents during cochlear development [5]. In addition, a human-specific pattern was reported for myelination of spiral ganglion neurons [6]. Despite these inter-species differences in cochlear development between rodents and humans, knowledge regarding cochlear development in humans is scarce because of ethical issues in obtaining human fetal tissues.

To bridge these inter-species differences between rodents and humans, the common marmoset (*Callithrix jacchus*), a primate model, has been exploited and investigated for cochlear development [7–9]. This animal model has also been used in studying deafness-related genes [10–14] and presbycusis [15], as well as in other areas of neuroscience [16]. These previous reports on the primate model have demonstrated the usefulness of the animal model in research on hearing.

In particular, previous reports have shown that cochlear development in this model differs from that of rodent models [7, 8]. For example, while Na–K-Cl cotransporter 1 (NKCC1) is well-known as a transporter of the stria vascularis and lateral wall, a distinct transient expression of NKCC1 in the organ of Corti during development has been observed in the common marmoset [7]. Distinct and complex changes in the expression patterns of synaptic vesicle exocytosis-related genes in hair cells during the developmental process of this primate have been reported [8]. However, neither have been reported in rodent models.

Previous embryological studies in this primate have focused only on the fetus after embryonic day 96 (E96), a relatively late stage of cochlear development [7–9]. Therefore, early development (before E96) of the cochlea in this animal model has not thoroughly been investigated, although several critical developmental processes are known to occur at this early stage. For example, when cochlear duct coils are formed or how sensory epithelial differentiation occurs in this animal are not well understood.

This study examined the cochlea from E70 to E92 of the common marmoset. We investigated important cochlear developmental steps, including formation of the coiled structure, specifications of the sensory epithelium, differentiation of hair cells, and development of neurons.

## Methods

### Specimens

Cadaverous temporal bone samples of common marmosets at E70 (*n* = 4), E77 (*n* = 2), E87 (*n* = 4), and E92 (*n* = 3) were used in this study. The animal experiments were approved by the Animal Experiment Committee of Keio University (number: 11006, 08,020) and were performed in accordance with the guidelines of the National Institutes of Health and the Ministry of Education, Culture, Sports, Science, and Technology of Japan.

### Tissue preparation

The temporal bone was dissected and fixed with 4% paraformaldehyde in phosphate-buffered saline (PBS) overnight immediately after euthanasia. Specimens were embedded in Tissue-Tek O.C.T. compound (Sakura Finetechnical Co., Ltd., Tokyo, Japan) for cross-sectioning. Seven-micrometer sections were used for immunohistochemical analysis.

### Immunohistochemistry

After a brief wash with PBS, the sections were heated (80 °C) in 10 µM citrate buffer (pH 6) for 15 min. After another brief wash, the sections were pre-blocked for 1 h at room temperature in 10% normal serum in PBS, incubated overnight with primary antibodies at 4 °C, and then incubated with Alexa Fluor-conjugated secondary antibodies for 60 min at room temperature. Nuclei were counterstained with Hoechst 33,258.

### Antibodies

The primary antibodies used in this study are listed in Table 1. The following secondary antibodies were used: goat anti-rabbit IgG, Alexa Fluor Plus 555 (A32732, 1:500, Invitrogen), goat anti-rabbit IgG, Alexa Fluor Plus 647 (A32733, 1:500, Invitrogen), goat anti-mouse IgG, Alexa Fluor Plus 488 (A32723, 1:500, Invitrogen), goat anti-mouse IgG1, Alexa 488 (A21121, 1:500, Invitrogen), goat anti-mouse IgG2b, Alexa 647 (A21242, 1:500, Invitrogen), goat anti-chicken IgY, Alexa Fluor 555 (A32932, 1:500, Invitrogen), donkey anti-mouse IgG, Alexa Fluor Plus 488 (A32766, 1:500, Invitrogen), donkey anti-chicken IgY, and Alexa Fluor 647 (703–605-155, 1:500, Jackson Immuno-Research).

**Table 1:** Primary antibodies used in this study

Antibody	Host	Isotype	Catalog ID	Provider	Dilution
Anti-POU4F3	Mouse	IgG1	sc-81980	Santa Cruz Biotechnology, Santa Cruz, CA, USA	1:200
Anti-ATOH1	Rabbit	IgG	21,215–1	Proteintech, Rosemont, IL, USA	1:500
Anti-SOX2	Goat	IgG	AF2018	R&D, Minneapolis, MN, USA	1:200
Anti-CDKN1B	Mouse	IgG1	610,242	BD, Franklin Lakes, NJ, USA	1:200
Anti-JAG1	Rabbit	IgG1	ab109536	Abcam, Cambridge, UK	1:200
Anti-GATA3	Rabbit	IgG	HPA029731	Atlas Antibodies, Bromma, Sweden	1:100
Anti-SOX10	Mouse	IgG1	365,692	Santa Cruz Biotechnology, Santa Cruz, CA, USA	1:100
Anti-OTX2	Goat	IgG	AF1979	BD, Franklin Lakes, NJ, USA	1:500
Anti-ISL1	Rabbit	IgG	ab109517	Abcam, Cambridge, UK	1:300
Anti-PAX2	Rabbit	IgG	901,002	Biolegend, San Diego, CA, USA	1:200
Anti-OTX1	Mouse	IgG1	Otx-5F5	DSHB, Iowa City, IA, USA	1:200
Anti-DLX5	Goat	IgG	sc-18152	Santa Cruz Biotechnology, Santa Cruz, CA, USA	1:100
Anti-PAX8	Mouse	IgG1	ACR438A	Biocare Medical, Pacheco, CA, USA	1:100
Anti-TUBB3	Mouse	IgG2b	GTX631836	GeneTex, Irvine, CA, USA	1:1000
Anti-NEUROD1	Goat	IgG	sc-1084	Santa Cruz Biotechnology, Santa Cruz, CA, USA	1:200
Anti-POU4F1	Mouse	IgG2b	sc-8429	Santa Cruz Biotechnology, Santa Cruz, CA, USA	1:200
Anti-MAFB	Rabbit	IgG	HPA005653	Atlas Antibodies, Bromma, Sweden	1:200
Anti-PRPH	Chick	IgY	ab39374	Abcam, Cambridge, UK	1:500
Anti-S100B	Rabbit	IgG	ab52642	Abcam, Cambridge, UK	1:200
Anti-RBFOX3	Mouse	IgG1	MAB377	Merck Millipore, Burlington, MA, USA	1:200

## Results

### Early morphological development of the common marmoset cochlea

**Fig. 1:**
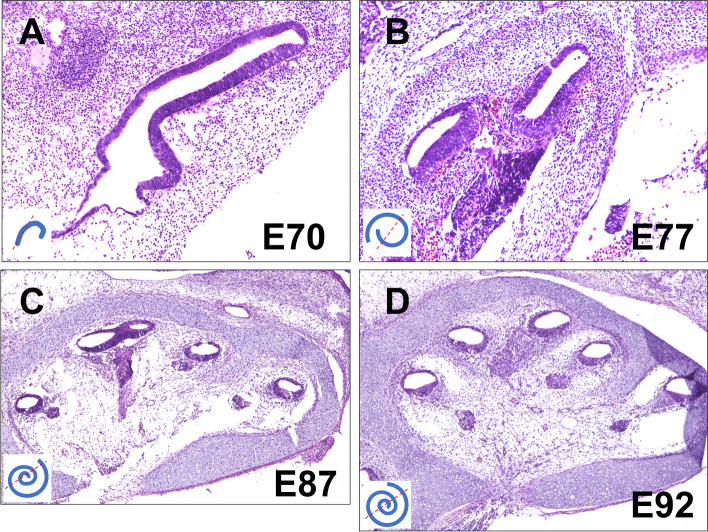
Histological analysis of the developing cochlea in the common marmoset using hematoxylin–eosin staining. (**A**) A cross-sectional view of the common marmoset cochlear duct in an E70 embryo. Cochlear duct formation had already started at E70. However, the coiled structure appeared only as a hook-like structure. (**B**) A cross-sectional view of the common marmoset cochlea duct in an E77 embryo. At E77, the elongation and coiling of the cochlear duct were more prominent. At this stage, the apical and basal turns can be distinguished. On the modiolar side, spiral ganglion neuron formation was observed at this stage. (**C**) A cross-sectional view of the common marmoset cochlea duct in an E87 embryo. At E87, the overall coiled structures of the cochlear duct were well developed and approximately two turns of the coiled structure were observed. The scala vestibuli and the scala tympani were not formed. (**D**) A cross-sectional view of the common marmoset cochlea duct in an E92 embryo. At E92, the coiled structure of the cochlear duct was almost completely formed. At this stage, the immature scala vestibuli and scala tympani were observed in the basal turns

The common marmoset has a gestation period of approximately 143–150 days [17]. Previously, we have reported that cochlear duct formation was complete, with apical, middle, and basal turns, in E96 embryos of the common marmoset. In this study, we first examined morphological changes in the cochlea during early development in the common marmoset using hematoxylin–eosin staining (Fig. 1).

In E70 embryos of the common marmoset, the cochlear duct appeared as a hook-like structure, and the coiled structure was incomplete (Fig. 1A). In E77 embryos, at least a one-turn coiled structure was observed (Fig. 1B). At this stage, a premature bony capsule of the cochlea was also observed. In E87, the cochlear duct was elongated, and a two-turn coiled structure was observed (Fig. 1C). At this stage, the structures of the cochlear bony capsules became more prominent. In E92, an almost entirely elongated coil was formed and a two-and a half-turn coiled structure was observed (Fig. 1D). Our observations indicated that the coiled structure of the common marmoset formed before E70 and its elongation continued until E92.

### Development of hair cells

Next, we investigated the initial formation of hair cells by examining the expression of hair cell markers, POU4F3 (POU class 4 homeobox 3) [18] and ATOH1 (atonal bHLH transcription factor 1) [19, 20].

**Fig. 2:**
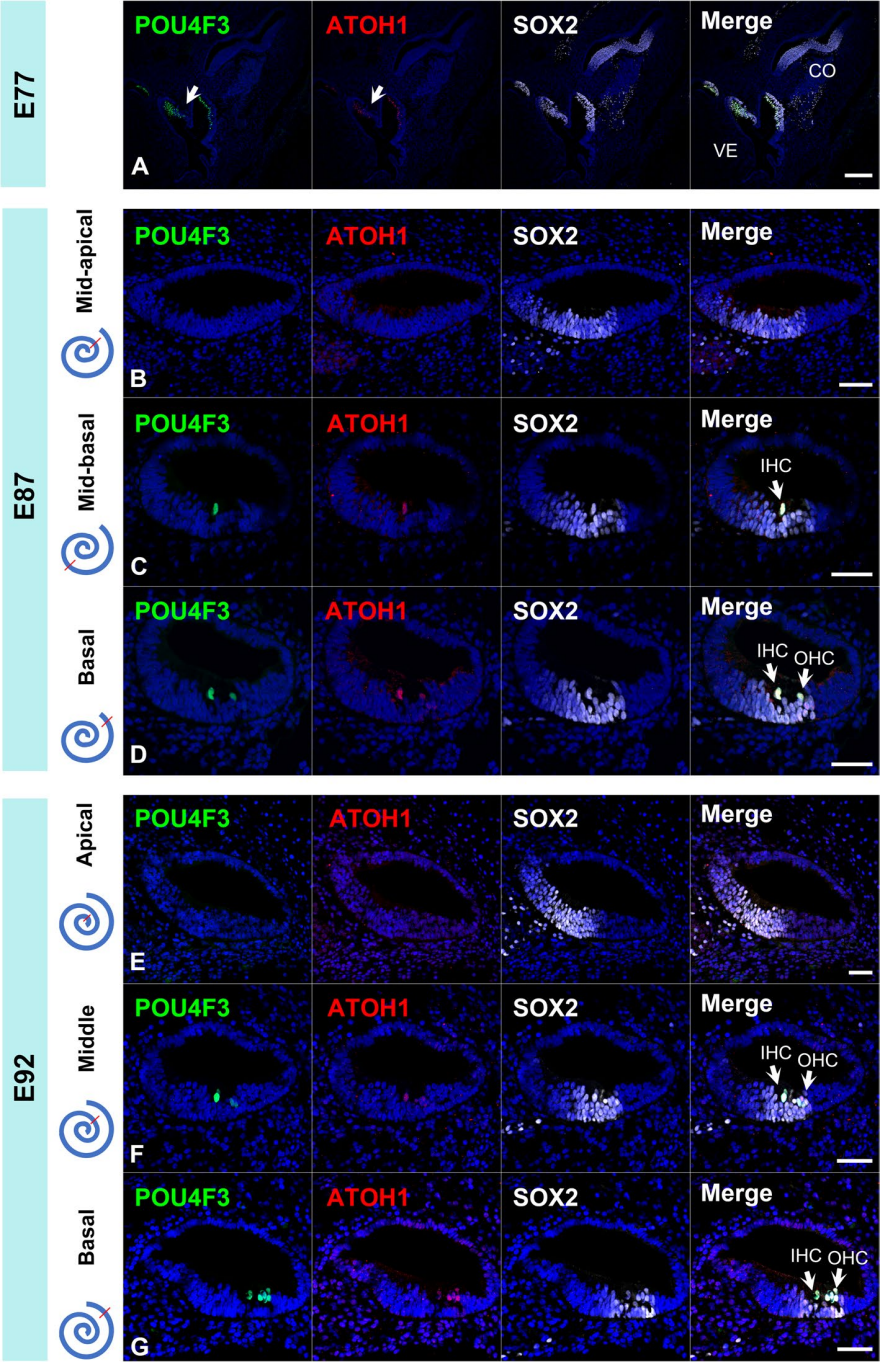
Expression of POU4F3 and ATOH1 in the early developing cochlea of the common marmoset. (**A**) In E77, no expression of POU4F3 and ATOH1 was detected in the cochlear, while expression of POU4F3 or ATOH1 was detected in the vestibular hair cells (arrow in A). (**B-D**) In E87, expression of POU4F3 and ATOH1 was detected in the basal and mid-basal turn. No expression of POU4F3 or ATOH1 was detected in the apical turn. In the basal turn, expression of POU4F3 and ATOH1 was detected in the inner hair cells and one row of the outer hair cells, while in the mid-basal turn, their expression was observed only in the inner hair cells. (**E–G**) In E92, POU4F3 and ATOH1 expression was observed in the middle and basal turns. No expression of POU4F3 or ATOH1 was detected in the apical turns. POU4F3 and ATOH1 expression was observed in both the inner and outer hair cells in both the middle and basal turns. Nuclei were counterstained with Hoechst stain (blue). Scale bar: 200 µm in A, 50 µm in B-G. VE, vestibular organ; CO, cochlea; IHC, inner hair cells; OHC, outer hair cells

In common marmosets, neither POU4F3 nor ATOH1 expression was observed in the E77 cochlear duct, while both of their expression was observed in the vestibular organ (Fig. 2A). In E87, expression of both POU4F3 and ATOH1 was observed in the SOX2 (SRY-box transcription factor 2) positive-prosensory domain (Fig. 2B-D). In the E87 cochlear duct, one row of inner hair cells was observed between the mid-basal and basal turns. In the basal turn, the development of outer hair cells was observed at this stage. In E92, hair cell development proceeded more apically, and both inner and outer hair cell development was observed in the middle turns (Fig. 2E-G). In contrast, no expression of hair cell markers was observed in the apical turns at this stage. These observations indicated that hair cell formation in the common marmoset started between E77 and E87 from the basal turns and progressed from the base to the apex.

### Specification of the prosensory domain in the developing common marmoset cochlea

Next, we investigated the timing of the specification of the prosensory domain in this animal using the cochlear prosensory domain markers, SOX2 [21, 22], CDKN1B (cyclin dependent kinase inhibitor 1B) [23], and JAG1 (jagged canonical Notch ligand 1) [23–25].

**Fig. 3:**
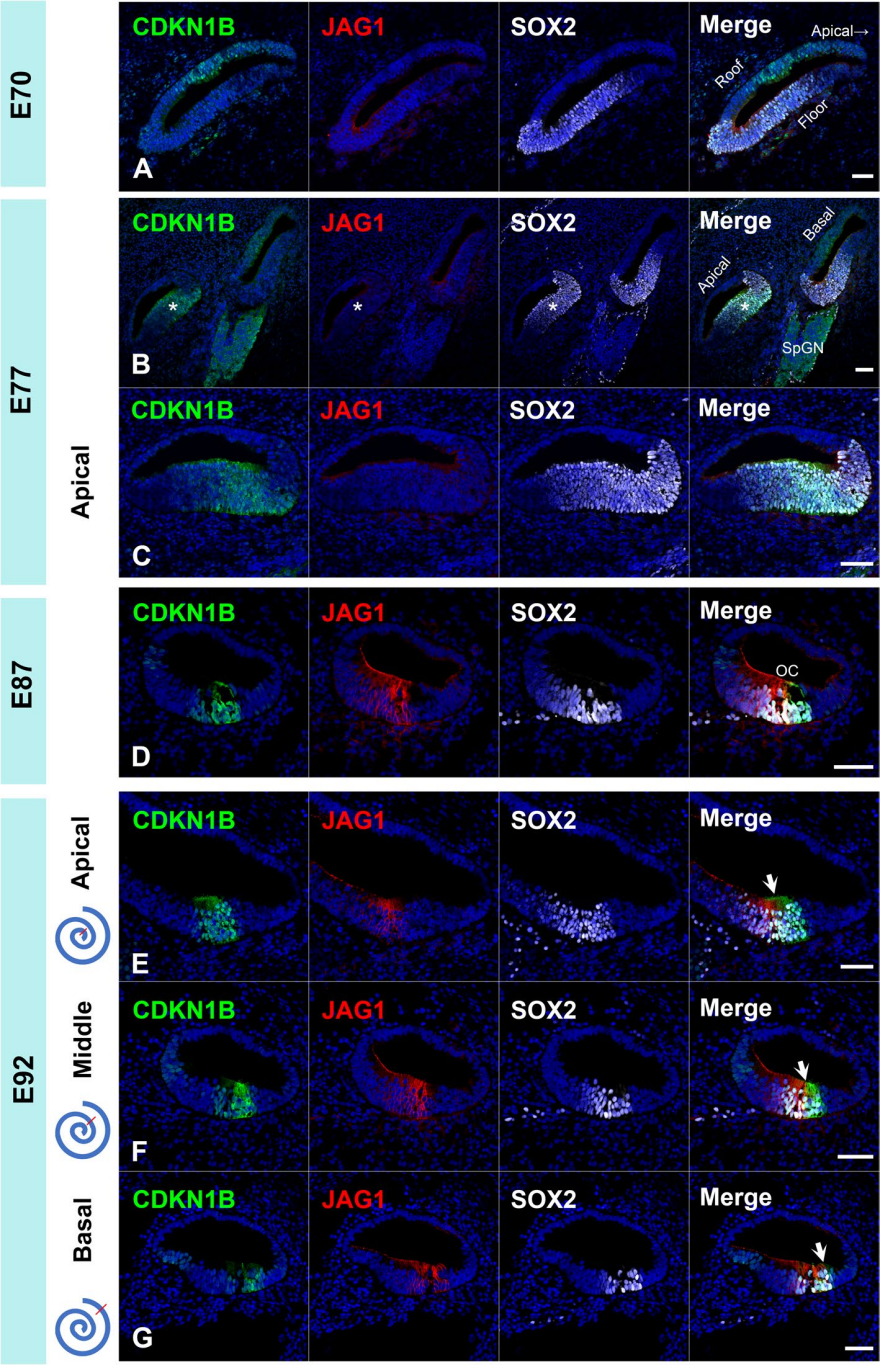
Sensory epithelium specifications of the cochlea of the common marmoset. (**A**) In E70, CDKN1B and JAG1 expressions were not observed on the floor of the cochlear duct. (**B** and **C**) In E77, CDKN1B expression was detected in the floor of the elongating cochlear duct and in the apical turns (asterisk in **B**). At this stage, no JAG1 expression was detected in the cochlear duct. In the apical turn, CDKN1B expression was observed in a part of the SOX2-positive regions (**C**). (**D**) In E87, JAG1 expression was observed in the cochlea. (**E–G**) In E92, both CDKN1B and JAG1 expression was observed in the cochlea. JAG1 expression was observed in the medial side of the SOX2-positive region in the apical turn (Arrow in **E**). In contrast, JAG1 expression was detected more laterally and broadly in the basal turn (Arrow in **F** and **G**). The nuclei were counterstained with Hoechst (blue). Scale bar: 50 µm. OC: organ of Corti

**Fig. 4:**
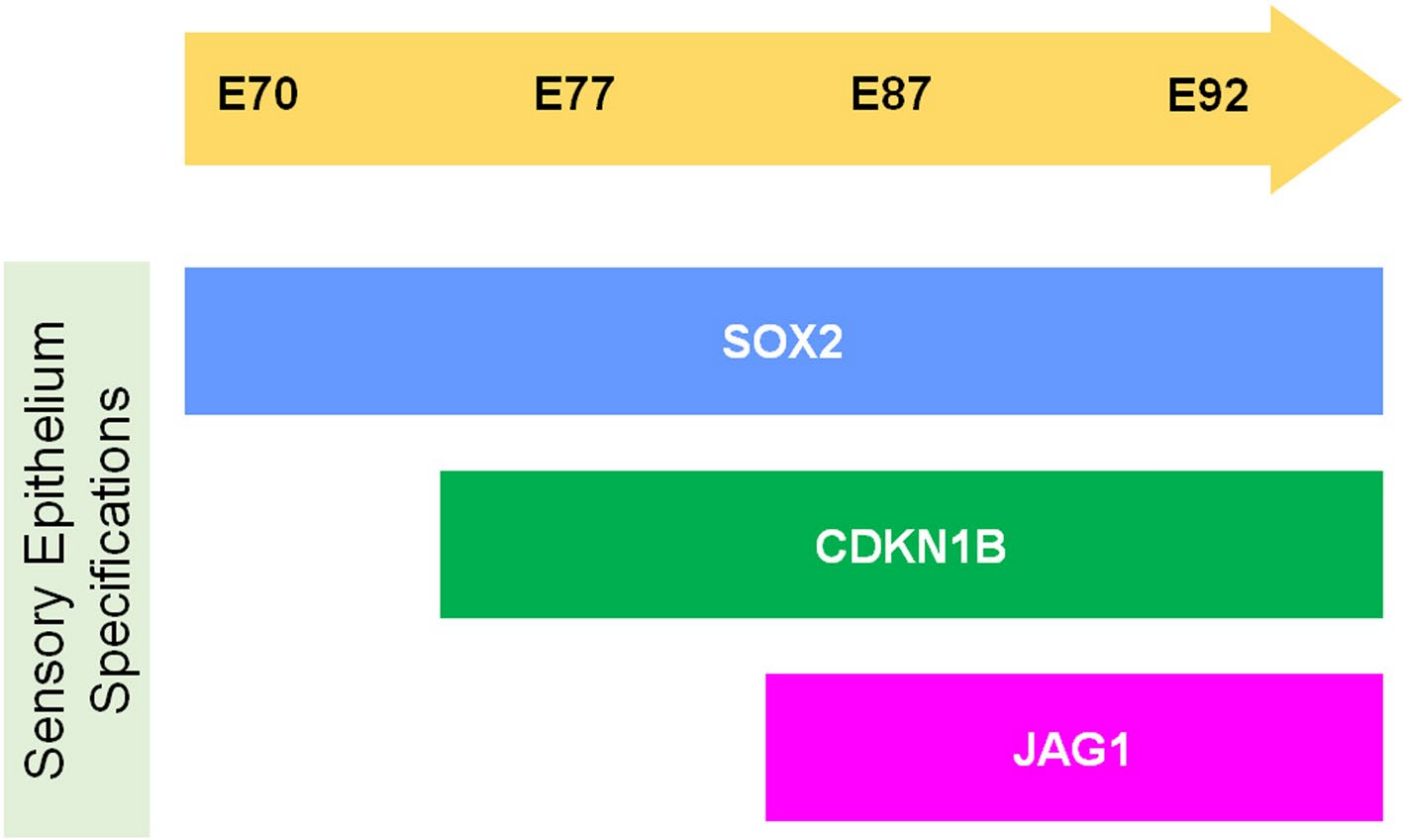
Schematic diagram showing expression of SOX2, CDKN1B, and JAG1. CDKN1B expressions in the SOX2-positive regions were observed at E77, while no JAG1 expression in the SOX2-positive regions was observed at this stage. At E87, both CDKN1B and JAG1 expression were observed in the SOX2-positive regions. This observation indicated that JAG1 expression followed CDKN1B expression in developing common marmoset cochlea

Ventral expression of SOX2 in the cochlear duct was observed in E70; however, CDKN1B and JAG1 were not observed in these SOX2-positive cells (Fig. 3A). In E77, CDKN1B and SOX2 double-positive cells were observed in the apical turns of the cochlear duct, but JAG1 expression was not observed at this stage (Fig. 3B and C). In E87, JAG1 expression was observed in the medial-ventral region of the cochlear duct (Fig. 3D). These observations indicated that the prosensory domain specification in this animal started at around E77 at the apical turn and spread to the basal turn. In E92, JAG1 expression was observed in the medial side of the SOX2-positive region in the apical turn, whereas it was broadly distributed in the SOX2-positive region in the basal turn (Fig. 3D-F). These expression patterns of SOX2 and JAG1 with apical to basal gradients were similar to those previously reported in mice [25–27]. In summary, as shown in the schematic diagrams in Fig. 4, CDKN1B expression in the SOX2- positive region preceded JAG1 expression in the common marmoset cochlear sensory epithelium specifications.

### Early patterning of the cochlear duct of the common marmoset

**Fig. 5:**
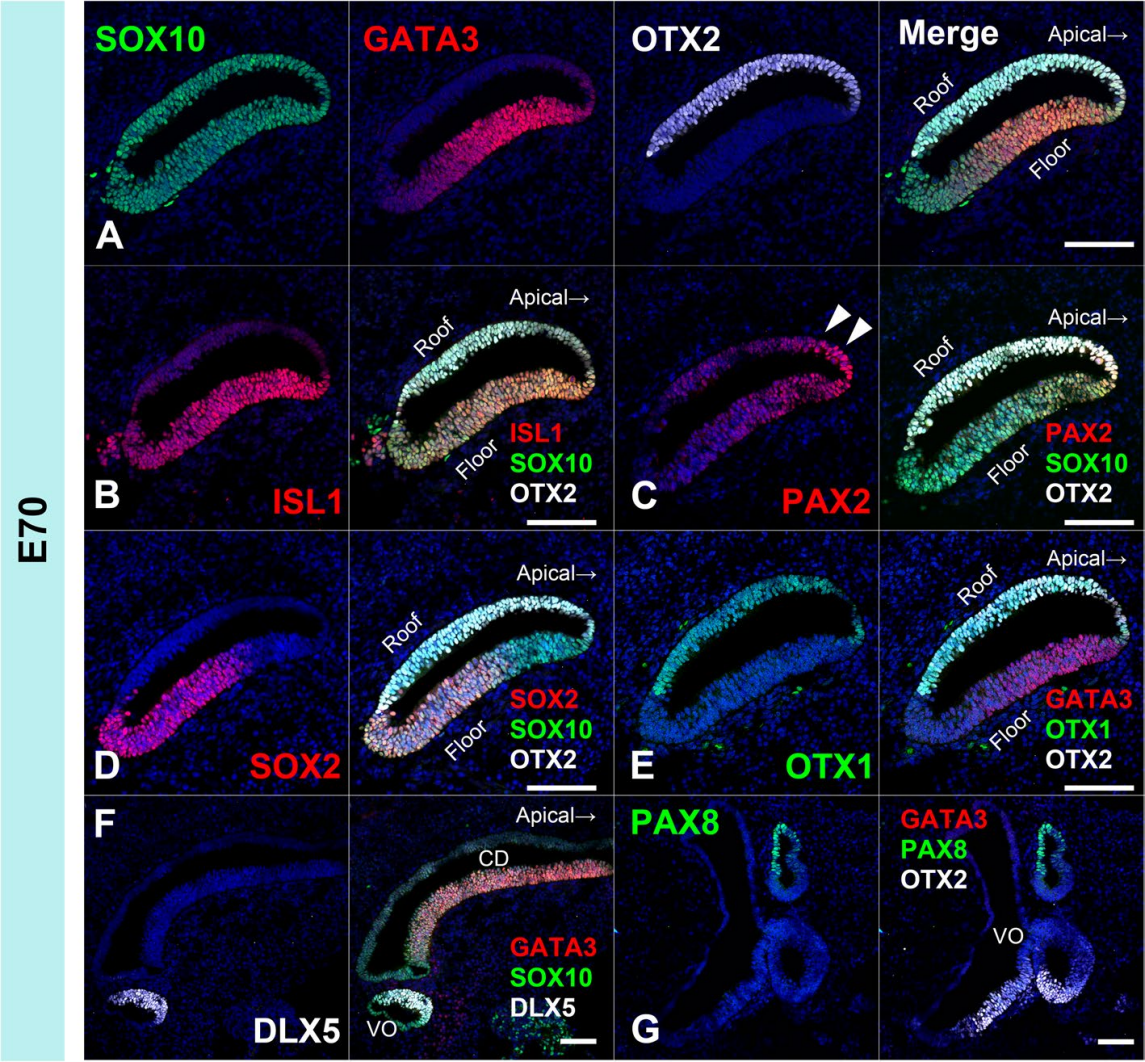
Expression patterns of OTX1, OTX2, PAX2, GATA3, SOX2, and ISL1 in E70 cochlea. (**A**) GATA3 expression was observed in the ventral portion of the cochlear duct at E70, while OTX2 expression was detected in the dorsal portion. (**B**) ISL1 expression was observed in the ventral portion of the cochlear duct. (**C**) PAX2 expression was detected in the lateral portion of the cochlear duct (arrowhead). (**D**) SOX2 expression was observed in the ventral portion and no overlapping expression with OTX2 was detected. (**E**) OTX1 expression was detected in the dorsal part and no overlapping expression with GATA3 was observed. (**F** and **G**) Expression of PAX8 and DLX5 was not detected in the cochlear duct at this stage. The nuclei were counterstained with Hoechst (blue). Scale bar: 100 µm. CD: cochlear duct, VO: vestibular organ

**Fig. 6:**
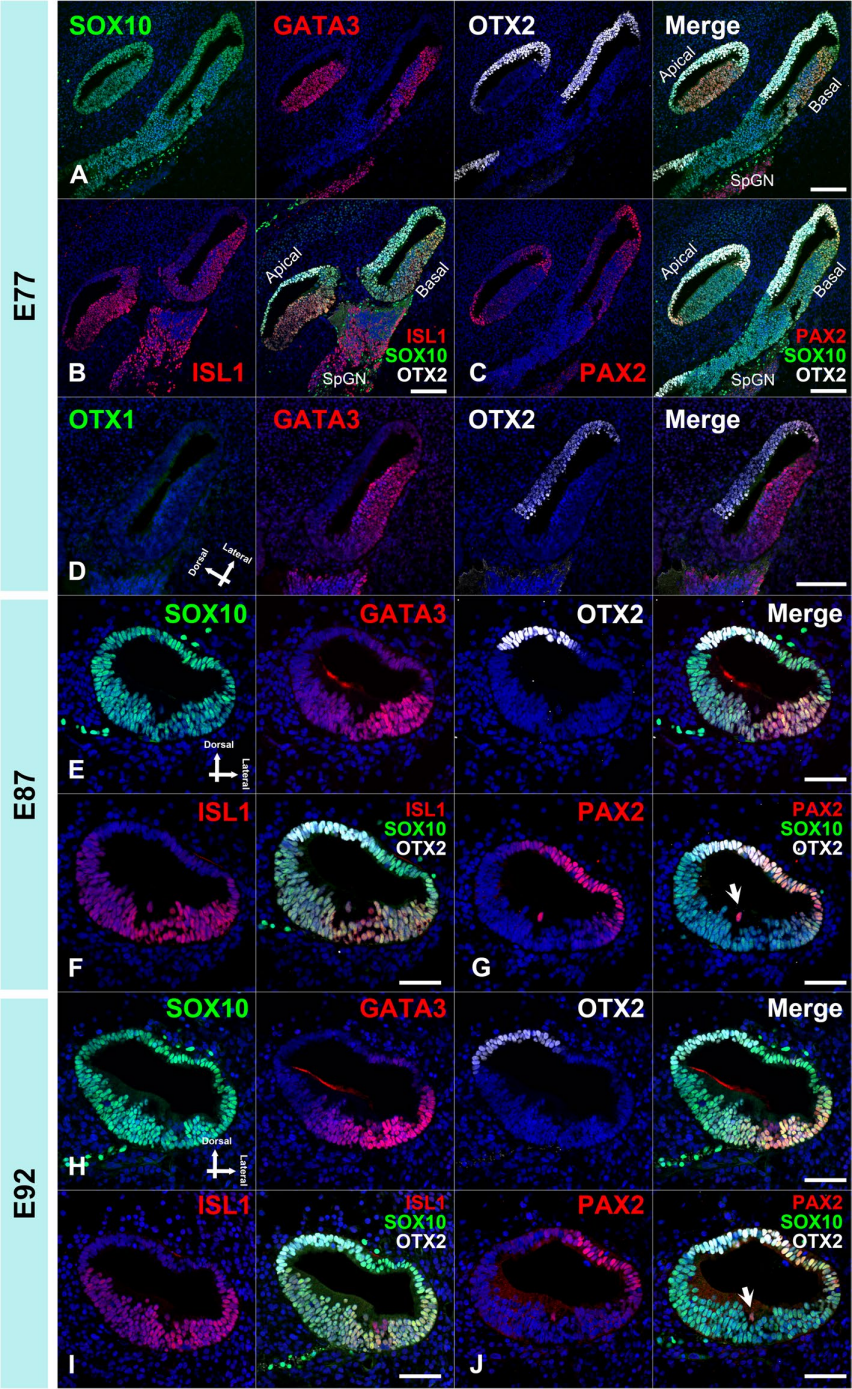
OTX2, PAX2, GATA3, and ISL1 expression patterns in E77, E87, and E92 cochlea. (**A-D**) Expression patterns of E77 cochlea. GATA3 and OTX2 expression was observed in the ventral and dorsal portion of the cochlear duct at E77, respectively (A). In the cochlear duct, ISL1 and PAX2 expression was observed in the ventral (**B**) and lateral portion (**C**), respectively. OTX1 expression diminished and no expression was detected in E77 (**D**). (**E–G**) Expression patterns in E87 cochlea. GATA3 and OTX2 expression was observed in the ventral and dorsal portion of the cochlear duct, respectively (**E**). ISL1 expression was observed in the ventral portion of the cochlear duct (F). PAX2 expression was detected in the lateral portion of the cochlear duct and hair cells (arrow in **G**). (**H-J**) Expression patterns in E92 cochlea. GATA3 and OTX2 expression was observed in the ventral and dorsal portion of the cochlear duct, respectively (**H**). ISL1 expression was observed in the ventral portion of the cochlear duct (**I**). PAX2 expression was detected in the lateral portion of the cochlear duct and hair cells (arrow in **J**). The nuclei were counterstained with Hoechst (blue). Scale bar: 100 µm in A-D, 50 µm in E-J. SpGN: spiral ganglion neuron

Early patterning of the cochlear duct, such as the organ of Corti, stria vascularis, and Reissner's membrane, is essential for the subsequent development of each compartment of the cochlear duct. This pattern of cochlear development is known to be fine-tuned by several genes [28–30]. Next, we evaluated the early patterning of the cochlear duct in this animal model by examining the expression of several markers, including OTX1 (orthodenticle homeobox 1) [31] and OTX2 (orthodenticle homeobox 2) [31] as dorsal markers, PAX2 (paired box 2) [32] as lateral markers, GATA3 (GATA binding protein 3) [33, 34] and ISL1 (ISL LIM homeobox 1) [35–37] as ventral markers (Fig. 5 and 6).

In E70 embryo of the common marmoset, GATA3 expression was observed in the ventral portion of the cochlear duct, while OTX2 expression was detected in the dorsal portion (Fig. 5A and D). ISL1 expression was observed in the ventral portion of the cochlear duct (Fig. 5B) and PAX2 expression was detected in the lateral portion of the cochlear duct (Fig. 5C). OTX1 expression was detected in the dorsal part (Fig. 5E). At this stage, expression of DLX5 and PAX8 was not detected in the cochlear duct (Fig. 5F and G).

In E77 cochlea, GATA3 and OTX2 expression was observed in the ventral and dorsal portion of the cochlear duct (Fig. 6A). In the cochlear duct, ISL1 and PAX2 expression was observed in the ventral and lateral portion (Fig. 6B and C). OTX1 expression diminished and no expression was detected in E77. In in E87 cochlea. GATA3 and OTX2 expression was observed in the ventral and dorsal portion of the cochlear duct (Fig. 6E). ISL1 expression was observed in the ventral portion of the cochlear duct (Fig. 6F). PAX2 expression was detected in the lateral portion of the cochlear duct and hair cells (Fig. 6G). In E92 cochlea, GATA3 and OTX2 expressions were observed in the ventral and dorsal portion of the cochlear duct (Fig. 6H). ISL1 expression was observed in the ventral portion of the cochlear duct (Fig. 6I). PAX2 expression was detected in the lateral portion of the cochlear duct and hair cells (Fig. 6J).

**Fig. 7:**
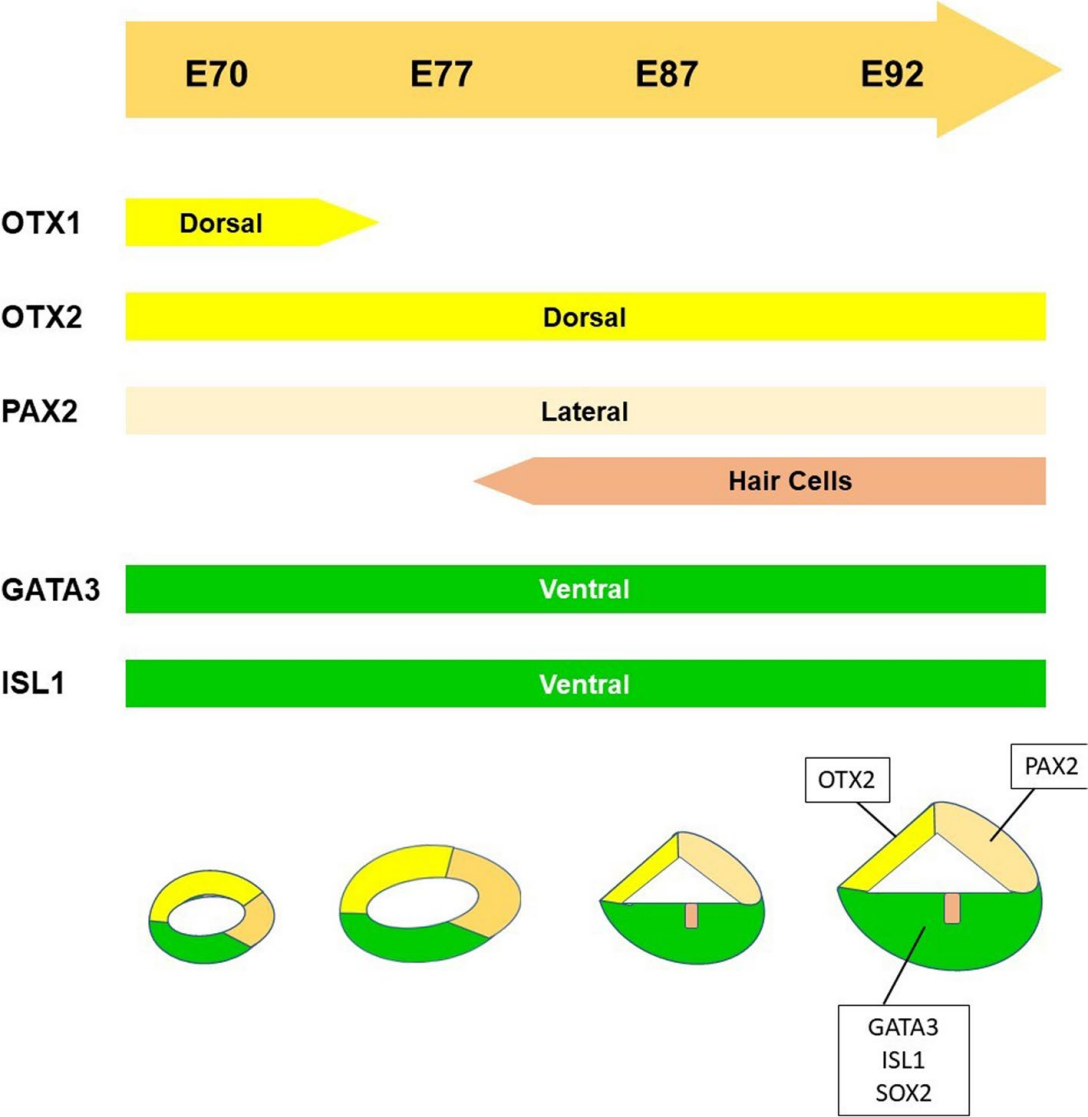
Schema showing expression patterns of OTX1, OTX2, PAX2, GATA3, and ISL1. Expression patterns of the genes that control early cochlear duct patterning were investigated. In common marmoset, OTX1 expression and OTX2 expression were observed in the dorsal portion of the cochlear duct, and PAX2 expression was observed in the lateral portion of the cochlear duct and hair cells. GATA3 expression and ISL1 expression were observed in the ventral portion of the cochlear duct

As shown in schematic diagrams, the expression patterns of these well-established conventional markers, which related to the early patterning of the cochlear development in the rodent models, were well conserved in the common marmoset cochlear development (Fig. 7).

### Early development of the spiral ganglion neuron of the common marmoset

Appropriate differentiation of spiral ganglion neurons and their innervation into the cochlear epithelium is essential for proper acquisition of hearing ability. This developmental process of the spiral ganglion neurons is controlled by the fine tuning of expression of transcription factors such as NEUROD1 (neuronal differentiation 1) [38–40], POU4F1 (POU class 4 homeobox 1) [41, 42], ISL1 [34, 36], GATA3 [34, 43], and MAFB (MAF bZIP transcription factor B) [44]. Next, we investigated early development of the spiral ganglion neuron of the common marmoset.

**Fig. 8:**
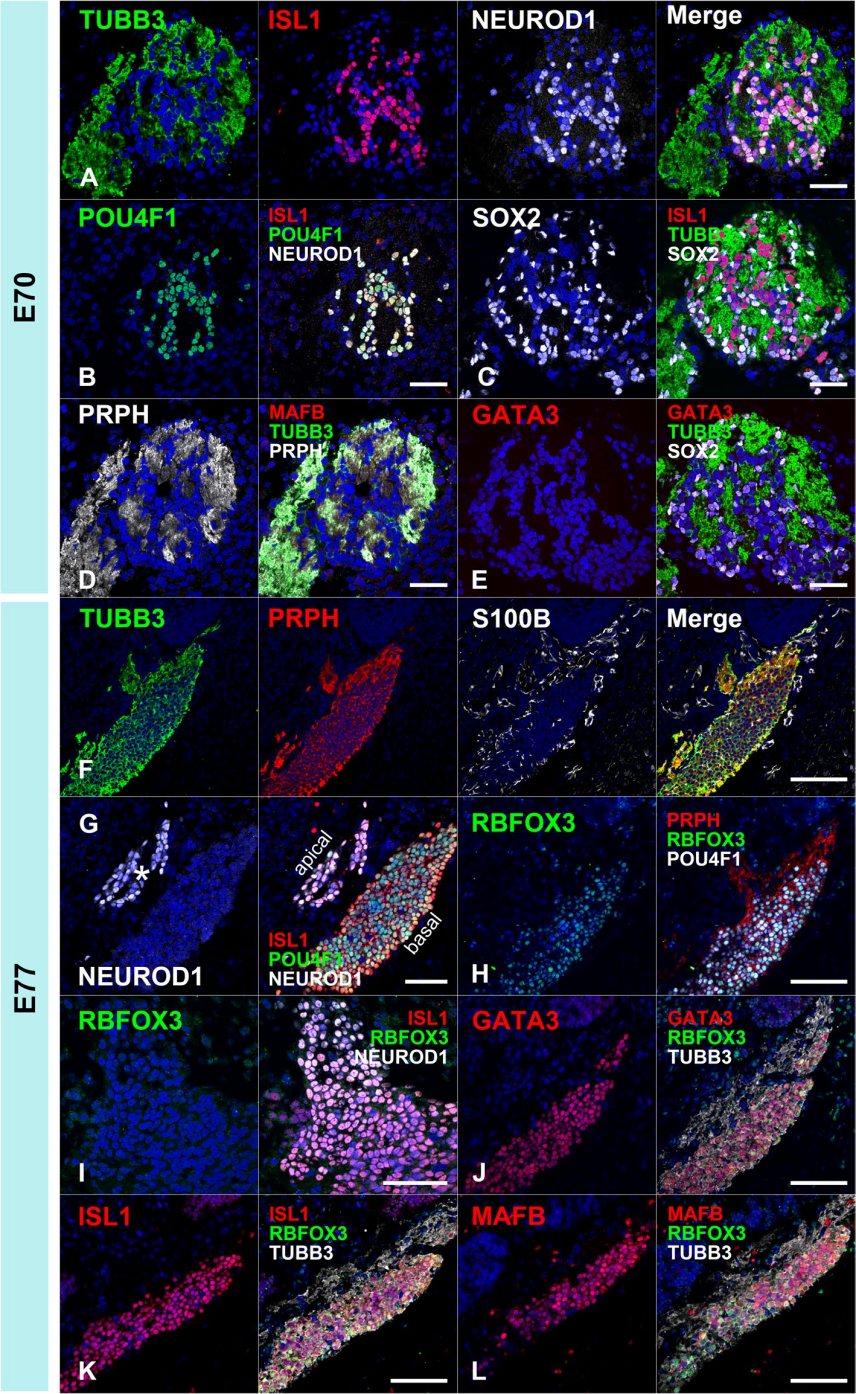
Cochlear neuronal development in E70 and E77. (**A-E**) Cochleovestibular ganglion in E70 cochlea. Expression of TUBB3, ISL1, and NEUROD1 was observed at E70 (**A**). Expression of POU4F1, SOX2, and PRPH was also observed in E70 cochleovestibular ganglion (**B**, **C**, and **D**). However, expression of MAFB and GATA3 was not detected in E70 cochleovestibular ganglion (**D** and **E**). (**F-L**) Spiral ganglion in E77 cochlea. Expression of TUBB3 and PRPH was observed in E77 spiral ganglion neurons (**F**). In E77, S100B-positive glial cells surrounding spiral ganglion neurons were observed (**F**). In E77, expression of NEUROD1 was observed in only an apical turn (asterisk in **G**), while RBFOX3 expression was observed in basal turns (**H**). No RBFOX3 expression was observed in the NEUROD1-positive spiral ganglion neurons (**I**). Expression of GATA3, ISL1, and MAFB was observed in the spiral ganglion neurons in E77 (**J-L**). The nuclei were counterstained with Hoechst (blue). Scale bar: 50 µm in A-E, G, and I, 100 µm in F, H, and J-L

**Fig. 9:**
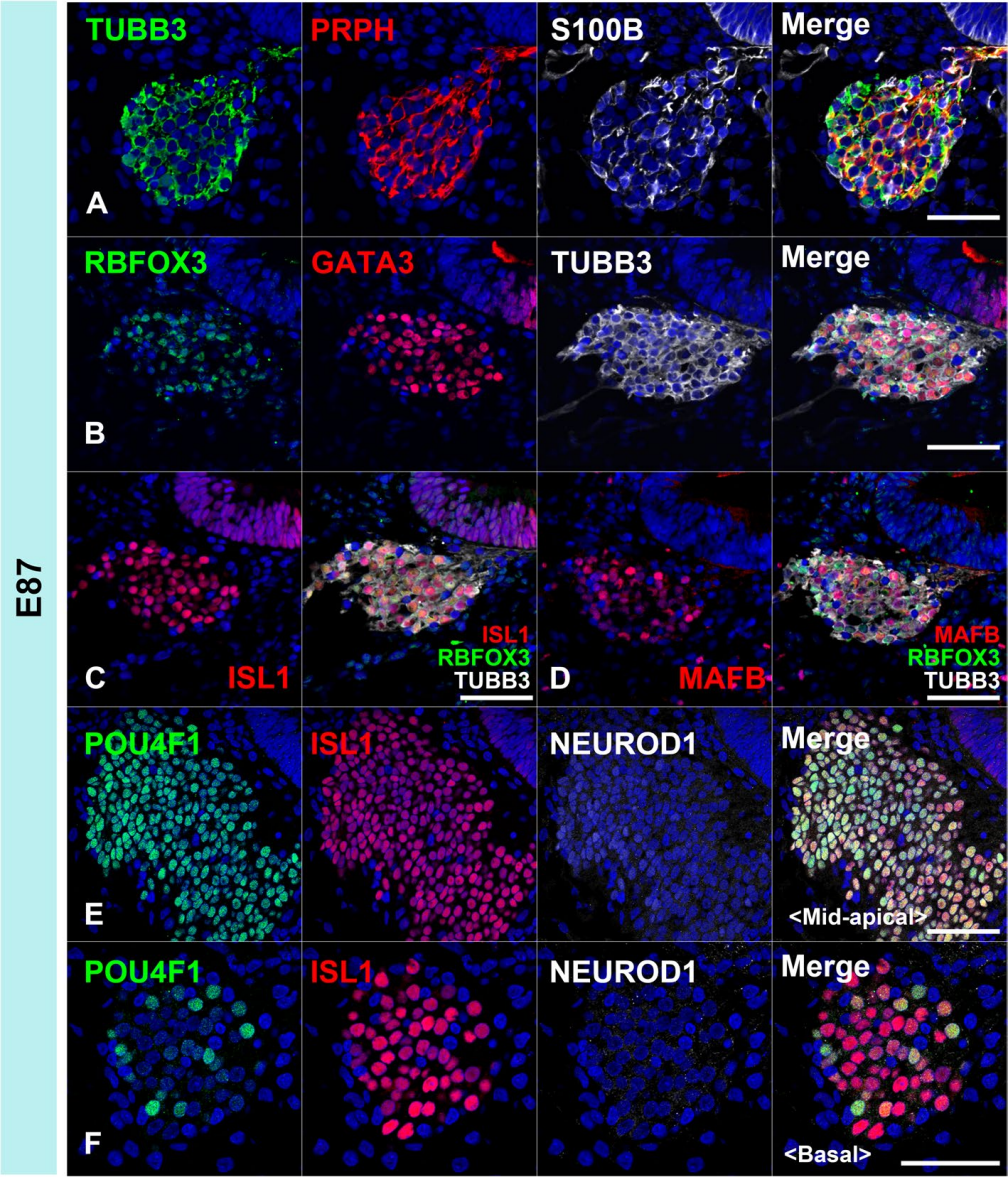
Spiral ganglion neurons in E87. Expression of PRPH3, RBFOX3, GATA3, ISL1, and MAFB was observed in the E87 spiral ganglion neurons (**A-D**). POU4F1 expression was observed in all spiral ganglion neuron turns, more in apical turns than in mid-apical turns. POU4F1 expression was observed in parts of the basal turns of spiral ganglion neurons. This indicated that the expression of POU4F1 decreased in basal turns. NEUROD1 expression was not detected in E87 spiral ganglion neurons (**E** and **F**). Nuclei were counterstained with Hoechst stain (blue). Scale bar: 50 µm

**Fig. 10:**
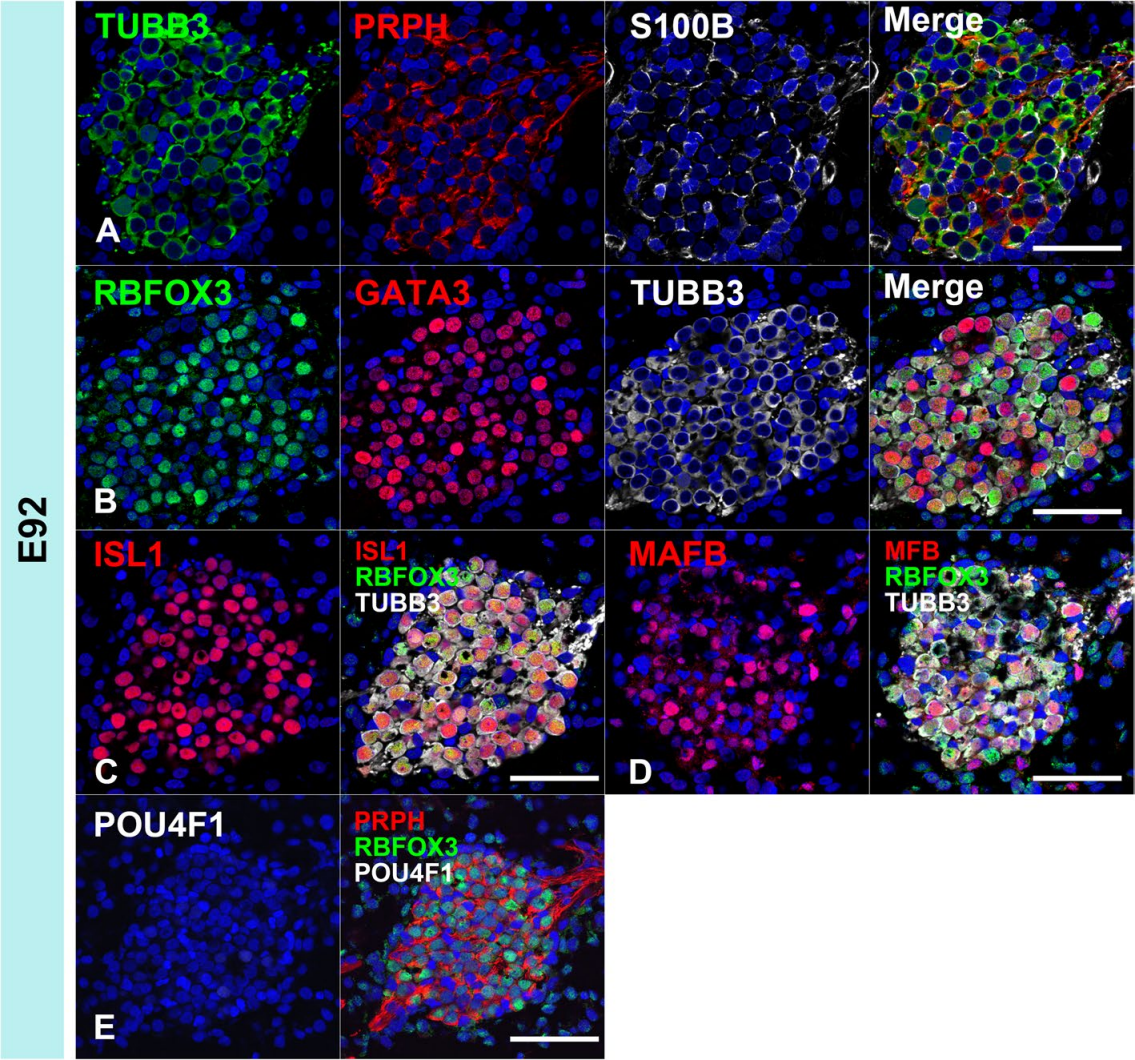
Spiral ganglion neurons in E92. Expression of PRPH3, RBFOX3, GATA3, ISL1, and MAFB was observed in the E92 spiral ganglion neurons (**A-D**). POU4F1 expression was not observed in the spiral ganglion neurons at this stage (**E**). Nuclei were counterstained with Hoechst stain (blue). Scale bar: 50 µm

In the common marmoset, cochleovestibular ganglion neurons were observed in the E70 cochlea (Fig. 8A-E). At this stage, the expression of TUBB3 and PRPH can be detected in cochleovestibular ganglion neurons (Fig. 8 A and D). Both NEUROD1 [38–40] and POU4F1 [41, 42] expression can be detected in the cochleovestibular ganglion neurons (Fig. 8B). In E70 embryo of the common marmoset, ISL1 expression was observed in the cochleovestibular ganglion neurons (Fig. 8C), whereas no expression of GATA3 and MAFB was detected (Fig. 8D and E). In the E77 cochlea (Fig. 8 F-L), spiral ganglion neurons come to be observed apparently and both NEUROD1 and POU4F1 were detected in the spiral ganglion neurons (Fig. 8G). At this stage, POU4F1 expression being apparent in most of the spiral ganglion neurons; however, NEUROD1 expression was restricted to neurons in the relatively apical turns (Fig. 8G). In contrast, RBFOX3 (RNA binding fox-1 homolog 3) expression was detected at E77 in NEUROD1-negative neurons located in the basal turns (Fig. 8H). After E77, ISL1, GATA3, and MAFB were observed in the spiral ganglion neurons (Fig. 8 J-L). At E87 (Fig. 9), although RBFOX3 expression was broader (Fig. 9B), NEUROD1 expression could not be detected in the spiral ganglion neurons (Fig. 9E and F). POU4F1 expression was reduced in the basal turns (Fig. 9F). At E92 (Fig. 10), RBFOX3 expression was detected broadly in the spiral ganglion neurons, whereas NEUROD1 and POU4F1 expression was not observed.

**Fig. 11:**
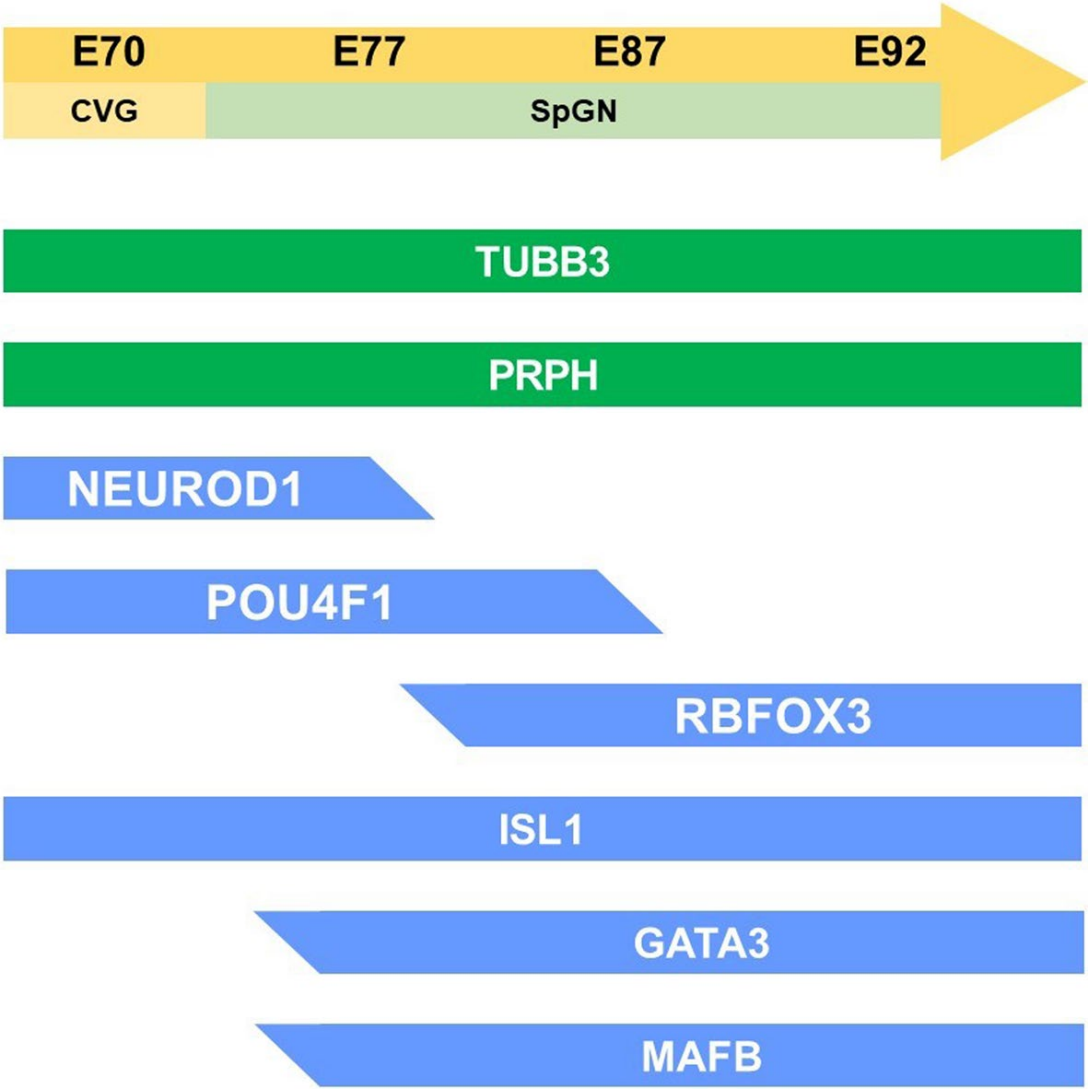
Schema showing the expression patterns of neuronal markers in early neuronal development. The expression patterns of genes involving the early development of spiral ganglion neurons were investigated. In the common marmoset, expressions of NEUROD1 and POU4F1 were observed in E77, and these expressions disappeared as development progressed. In contrast, broader RBFOX3 expression was observed after E87. GATA3 expression and MAFB expression followed ISL expressions in the spiral ganglion neuron of the common marmoset

As shown in the schematic diagram, changes of the expression patterns of the transcription factors, which are known to be involving neuronal development in mice, were also observed during the development and maturation of spiral ganglion neurons in the development of the common marmoset cochlea (Fig. 11).

### Integration of glial cells into the spiral ganglion neuron

Glial cells surrounding the spiral ganglion neurons originate from neural crest cells and migrate to the spiral ganglion [45, 46]. After integration of the migrating glial cells into the spiral ganglion, they differentiate to form myelin [47]. Previously, we have observed myelination of the spiral ganglion neurons at E115 [8]; however, migration and integration of glial cells in this primate have not been evaluated. Hence, we investigated the development of glial cells in the spiral ganglion using SOX10 (SRY-box transcription factor 10) and S100B (S100 calcium binding protein B), which have been used for the markers of glial cells derived from migrating neural crest cells in the spiral ganglion neurons [47]. Finally, we investigated the integration of glial cells into the spiral ganglion neurons.

**Fig. 12:**
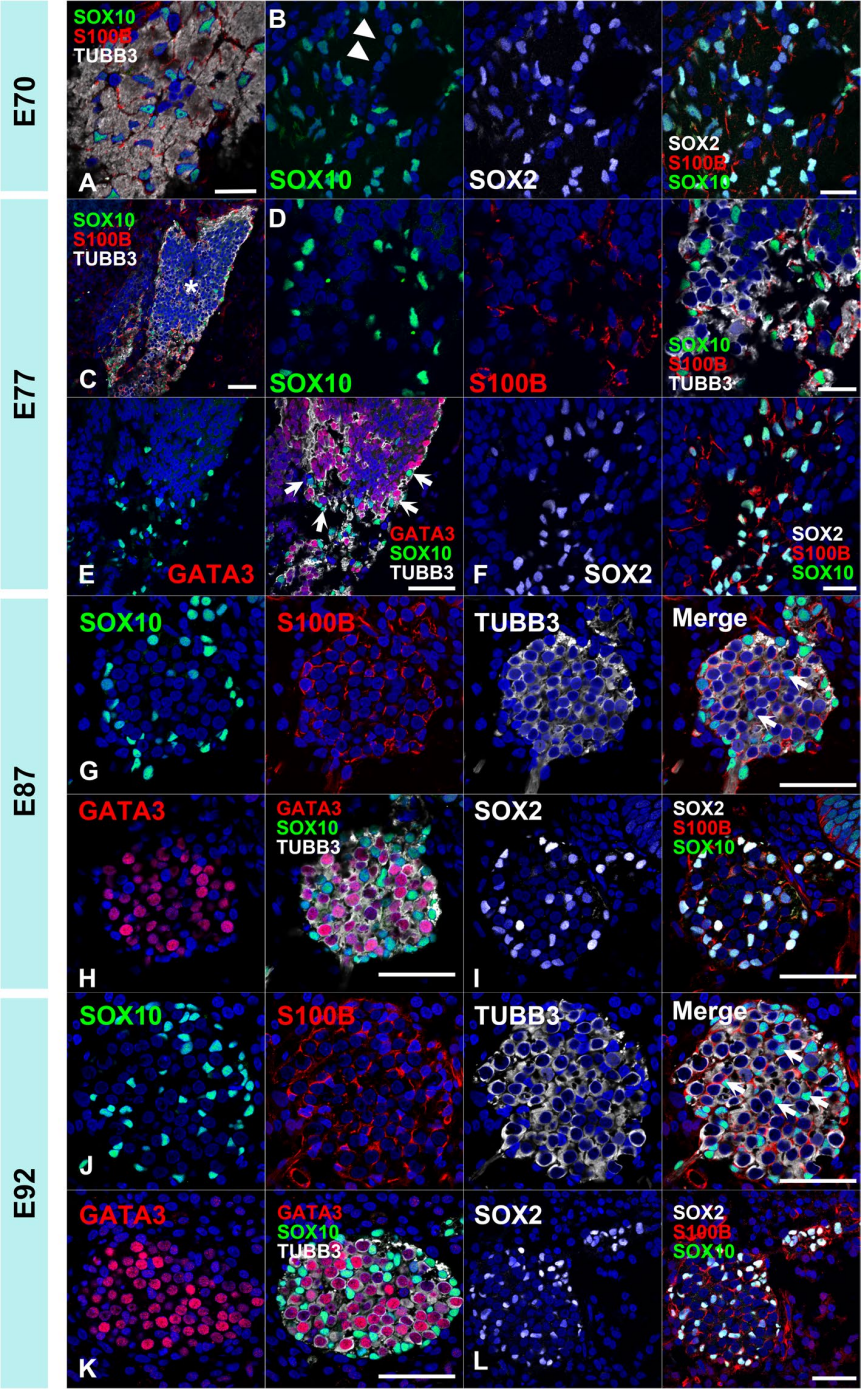
Early development and integration of the glial cells of the cochlear spiral ganglion. (**A** and **B**) Glial cells in the E70 cochleovestibular ganglion. SOX10^+^/S100B^+^ glial cells were observed in the E70 cochleovestibular ganglion (**A**). Notably, the small number of SOX10^−^/SOX2^+^/S100B^+^ glial cells were also observed at this stage (arrowhead in **B**). (**C-F**) Glial cells in the E77 spiral ganglion. At E77, glial cells were observed surrounding the spiral ganglion neurons at this stage (arrow in **E**). No SOX10-positive cells were observed in the center of the spiral ganglion (asterisk in **C**). At this stage, S100B-positive glial cells expressed both SOX2 and SOX10 (**F**). (**G-I**) Glial cells in E87. Several SOX10-positive cells were observed between the spiral ganglion neurons (arrow in **G**). (**J-L**) Glial cells in E92. Integration of the SOX10-positive glial cells in the spiral ganglion was more prominent. The number of SOX10-positive cells observed between the spiral ganglion neurons increased (arrow in **J**). The nuclei were counterstained with Hoechst (blue). Scale bar: 20 µm in A, B, D, and F. 50 µm in C, E, and G-L

In the common marmoset, SOX10^+^ /S100B^+^ glial cells [47] were observed at the E70 cochleovestibular ganglion (Fig. 12 A and B). This indicated that the neural crest cells migrated into the ganglion [45, 46] before E70 in this animal. At E77 (Fig. 12 C-F), many of the glial cells were located around the spiral ganglion neurons, while several cells were positioned between the neurons. The integration of satellite glial cells was completed at E87 and E92 (Fig. 12 G-L). At E70, SOX2 expression was observed in SOX10-negative cells in the cochleovestibular ganglion; At E77, SOX2 expression in the spiral ganglion was observed only in SOX10^+^/S100^+^ glial cells.

## Discussion

### Development of hair cells and specification of the prosensory domain in the developing common marmoset cochlea

**Fig. 13:**
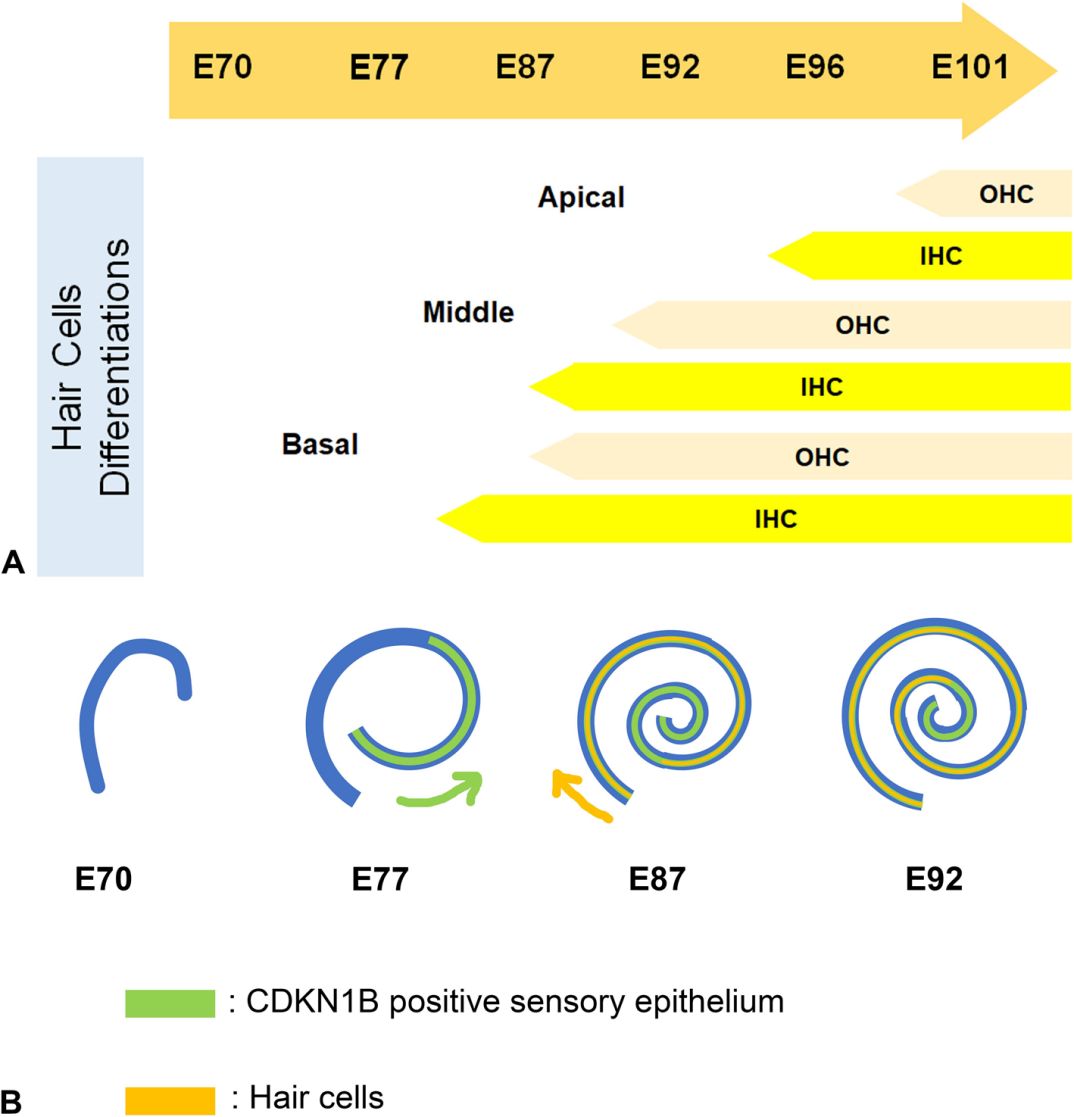
Schema showing early cochlear duct and sensory epithelium formation. (**A**) Schema showing differentiation of hair cells. Hair cells differentiated along the basal-to-apical axis and from inner to outer side. (**B**) Schema showing cochlear duct development. Cochlear duct elongation was observed between E70 and E92. CDN1B expression started from the apical turns in E77. In contrast, hair cells were observed in E87 in the basal turns. IHC, inner hair cells; OHC, outer hair cells

Development of hair cells and specification of the prosensory domain following the cochlear elongation is the one of the most essential steps in cochlear development. Our observation of the early cochlear development of the common marmoset identified the time points of these critical steps in this model animal. Combining observation in this study with our previous report, [7] hair cells developments of the common marmoset start between E77 and E87 from the basal turns and the progression reach to the apex turn at E101 (Fig. 13A). Preceding to this hair cells development, the elongation of the cochlear duct is observed until E92 (Fig. 13B).

In the human fetus, elongation of the cochlear duct starts at 4 − 5 weeks of gestation [48, 49], and the coiled cochlea can be observed at approximately 9 − 10 weeks of gestation [48–50]. In humans, specification of the sensory epithelium is observed at 9 weeks of gestation [51], and the differentiation of auditory hair cells begins at 12–14 weeks of gestation [5, 52]. Contrastingly, cochlear anlage becomes evident at E10.75 in mice, the first turn of the cochlea appears at E12, and the completely coiled cochlea is observed at E17 [53]. POU4F3 expression is observed at E15.5[18] and ATOH1 is expressed in a single row of cells from the base to the mid-base of the cochlea at E14.5 [19]. Combining the results of these previous reports with our present observations, we concluded that the E70 cochlea of the common marmoset was equivalent to the human cochlea at approximately 8 weeks of gestation and the E12 mouse cochlea. Similarly, the E87 cochlea of the common marmoset corresponded to the human cochlea at approximately 12 weeks of gestation and the E15 mouse cochlea.

Specification of the prosensory domain, which is essential for the subsequent formation of the organ of Corti, is an important step in cochlear development. Reports show that this specification developed during the elongation of the cochlear duct both in mice (E12.5 − E14.5) [22, 25, 54] and humans (9 weeks of gestation) [51]. Next, we investigated the timing of the specification of the prosensory domain in this animal using the cochlear prosensory domain markers, SOX2 [21, 22], CDKN1B [23], and JAG1[23–25]. This investigation revealed that prosensory domain specification was started at around E77 at the apical turn and spread to the basal turn until E87 in common marmoset (Fig. 13B).

Unlike that observed in mice, CDKN1B expression was followed by JAG1 expression during the specification of the cochlear prosensory domain in the common marmoset (Fig. 3 and 4). In mice, JAG1 expression is observed as early as in E12.5 and it overlaps with the SOX2 domain [25]. CDKN1b expression is observed at around E13.5–E14.5 [23, 25]. This determination of the prosensory domain via JAG1-mediated Notch1 signal activation occurs via lateral induction [26]. It is known that conditional knockout of *Jag1* in the cochlea results in downregulation of *Sox2* and *Cdkn1b*; therefore, prior expression of JAG1 is essential for the expression of SOX2 and CDKN1B in mice [25, 55]. However, prosensory domain specification occurred in this conditional knockout mouse, indicating the possibility of compensation of JAG1 by other Notch ligands [25].

Our observation that JAG1 expression was preceded by CDKN1B or SOX2 expression suggested that CDKN1B and SOX2 expression was controlled by other factors such as other Notch ligands, and that the role of JAG1 in controlling the expression of CDKN1B or SOX2 may be less dominant in this primate but needs validation. Until now, the expression patterns of JAG1 and CDKN1B in developing human fetuses have not been reported. Thus, we cannot conclude that this inter-species difference between common marmosets and mice will also be observed between humans and mice. However, our observations suggest the possibility of primate-specific control of prosensory domain specification of the cochlea. Nonetheless, further studies are warranted in the future.

Despite the difference in initial expression patterns and the lateral induction process, expression of JAG1, CDKN1B, and SOX2 in the later process of prosensory domain specifications are well conserved between common marmoset and mice [25–27]. In both mice at E14.5 and the common marmoset at E87, JAG1 was expressed mainly in the Kölliker's organ. Thus, JAG1 expression showed only a slight overlap with CDKN1B expression, and most of the prosensory domain, as assessed by CDKN1B expression, was JAG1-negative. Along with cochlear development, the JAG1-positive region progressed to the lateral side. In the later stages of development, Notch signaling is believed to divert the development of the prosensory domain into hair cells via lateral inhibition [26]. This preservation of expression pattern may suggest that lateral inhibition of cochlear development is well-preserved in mammals.

### Early patterning of the cochlear duct of the common marmoset

For investigating the early patterning of the cochlear duct, we used OTX1 and OTX2 as dorsal markers of the developing cochlear duct, GATA3 and ISL1 as ventral markers, and PAX2 as a lateral marker.

*OTX1* and *OTX2* encode members of the bicoid subfamily of homeodomain-containing transcription factors, which are required for the normal development of the inner ear [31]. In mouse cochlea, co-expression of OTX1 and OTX2 can be observed in the roof of the cochlear duct until E13, whereas OTX1 expression was not detected in the cochlea at E14 [31]. In contrast, OTX2 expression was observed in the non-sensory area of the roof of the cochlear duct, corresponding to the Reissner's membrane [56]. In the common marmoset, co-expression of OTX1 and OTX2 was detected in the E70 cochlea; however, after E77, only OTX2 expression was observed in the dorsal part of the cochlear duct.

*GATA3* encodes a protein that belongs to the GATA family of transcription factors. In the developing cochlea of mouse, GATA3 expression has been reported in the cochlear sensory epithelium and spiral ganglion neurons [33, 34]. In the cochlear duct of the E13.5 mouse, GATA3 expression was observed in the prosensory domain and the outer sulcus. *ISL1* encodes a member of the LIM/homeodomain family of transcription factors. In the developing cochlea of mouse, ISL1 expression has also been observed in the cochlear sensory epithelium and spiral ganglion neurons [35, 36]. Reports show that ISL1 expression is dispensable for the development of the mouse cochlear prosensory region [37]. In the common marmoset, we detected the expression of both GATA3 and ISL1 in the developing cochlear duct. Expression of both was observed in the ventral cochlear sensory epithelium during the periods examined in this study.

*PAX2* encodes a paired box protein, which is essential for normal inner ear morphogenesis [32]. In the developing cochlea of mouse, PAX2 expression was reported in the lateral wall of the cochlear duct and presumptive stria vascularis [32]. PAX2 expression in hair cells has also been reported in the developing cochlea of mice [43] and humans [57]. In the common marmoset, we detected the expression of PAX2 in the developing cochlear duct. Relatively broad PAX2 expression was detected in the E70 cochlea; however, PAX2 expression was restricted to the lateral wall of the cochlear duct in E77. In E87 and E97, PAX2 expression was observed on the lateral side of the cochlear epithelium and hair cells.

In addition, we examined the expression patterns of PAX8 (paired box 8) and DLX5 (distal-less homeobox 5). *PAX8* encodes a paired box protein and *DLX5* encodes a member of the homeobox transcription factor gene family. Both PAX8 and DLX5 are important for the specification of the otocyst and subsequent cochlear development [58, 59]. PAX8 expression has been reported in the otic placode, where it cooperates with PAX2 for inner ear morphogenesis and innervation in mice [58]. DLX5 expression has also been reported in developing otic placodes, and it is essential for the morphogenesis of the semicircular canal [59, 60]. In the common marmoset, we did not detect the expression of either PAX8 or DLX5 in the developing cochlear duct at E70, whereas they were expressed in some of the vestibular organs (Fig. 5F and G).

Although the expression patterns of genes investigated in this study were well conserved between the species, inter-species differences were observed in the expression patterns of genes related to prosensory domain specification that proceed simultaneously with this patterning.

### Early development of the spiral ganglion neuron of the common marmoset

So far, relatively large inter-species differences between rodents and primates have been reported with respect to the development of spiral ganglion neurons [5, 7]. For example, unlike in rodents [5, 61], in which PRPH expression is detected in relatively late stages of cochlear development, PRPH was expressed in spiral ganglion neurons prior to hair cell innervation in humans and common marmosets [5, 7]. In this study, we investigated the early development of spiral ganglion neurons in the common marmoset.

*NEUROD1*, a member of the neuroD family of basic helix-loop-helix (bHLH) transcription factors, is also known as one of the proneural genes responsible for the development of neuroectodermal progenitor cells. NeuroD expression has been reported in the developing mouse cochlea [39], and it is essential for neuronal differentiation of spiral ganglion neurons [38, 39]. In mice, NeuroD expression was detected in the spiral ganglion in E9.5, and its expression was observed until E13.5 [40]. *POU4F1* encodes a member of the POU-IV class of neural transcription factors, POU4F1, also known as brain-specific homeobox/POU domain protein 3A (BRN3A). POU4F1 regulates soma size, target field innervation, and axon pathfinding of spiral ganglion neurons [41]. In mice, POU4F1 expression was observed as early as E9.5, and it was downregulated in the spiral ganglion neurons in E17.5 [42]. *RBFOX3* encodes a member of the RNA-binding FOX protein family, which is involved in the regulation of alternative splicing of pre-mRNA. RBFOX3 is also known as the neuronal nuclei (NeuN) antigen, which has been widely used as a marker for post-mitotic neurons [62, 63]. RBFOX3 expression has been reported in post-mitotic mature spiral ganglion neurons after E11.5 in mice [42]. The sequential changes in the expression of NEUROD1, POU4F1, and RBFOX3 during cochlear development are well conserved between mice and common marmosets.

Studies have shown that ISL and GATA3 are essential for the proper development of spiral ganglion neurons and sensory epithelium [34]. In the mouse cochlea, *Isl1* expression was observed in the inner ear neuronal lineage from embryonic day E8.5 to E9.5, similar to the expression of NeuroD [36]. In contrast, at E9.5, GATA3 expression was detected only in the otic epithelium, and no expression was detected in the neuronal lineage [43]. GATA3 expression was first observed in the selected neuronal lineage at E12.5, which increased all over the spiral ganglion neurons after E13.5 [34]. Previous studies have shown that GATA3 is essential not for differentiation but for the survival of spiral ganglion neurons [34]. *MAFB* encodes MAFB, a basic leucine zipper (bZIP) transcription factor. MAFB acts downstream of GATA3 to control the differentiation of spiral ganglion neurons and drive the differentiation of the large post-synaptic density characteristic of the ribbon synapse [44]. The sequential expression patterns of ISL1, GATA3, and MAFB were conserved between mice and marmosets.

### Integration of glial cells into the spiral ganglion neuron

Finally, we compared the timing of integration of glial cells into the spiral ganglion neuron between the species. In mouse cochlea, SOX10-positive glial cells originating from neural crest cells were observed in the spiral ganglion neurons at E12.75. At this stage, the SOX10-positive glial cells wrapped the spiral ganglion neurons [64]. and also expressed SOX2. As development progressed, the SOX10-expressing cells were integrated throughout the spiral ganglion at E17.5 [64].

The satellite glial cells of the developing cochlea of the human fetus express S100B, which is a member of the S100 family of proteins containing two EF-hand calcium-binding motifs [5]. In humans, both SOX10- and S100B-positive glial cells can be observed in 9 weeks gestation fetuses [47], which integrate into the spiral ganglion neurons and envelope them between 12 and 14 weeks of gestation in humans [47].

In common marmoset, the integration of satellite glial cells was observed at E87 (approximately 12 weeks of gestation in human and the E15 mouse cochlea). Our observation indicated that glial cell integration of the common marmoset is more similar to human, and this integration was observed before hair cell differentiation in the primate. This timing was relatively earlier than that in mice [64].

### Inter-species difference of cochlear development between rodents and primates

**Fig. 14:**
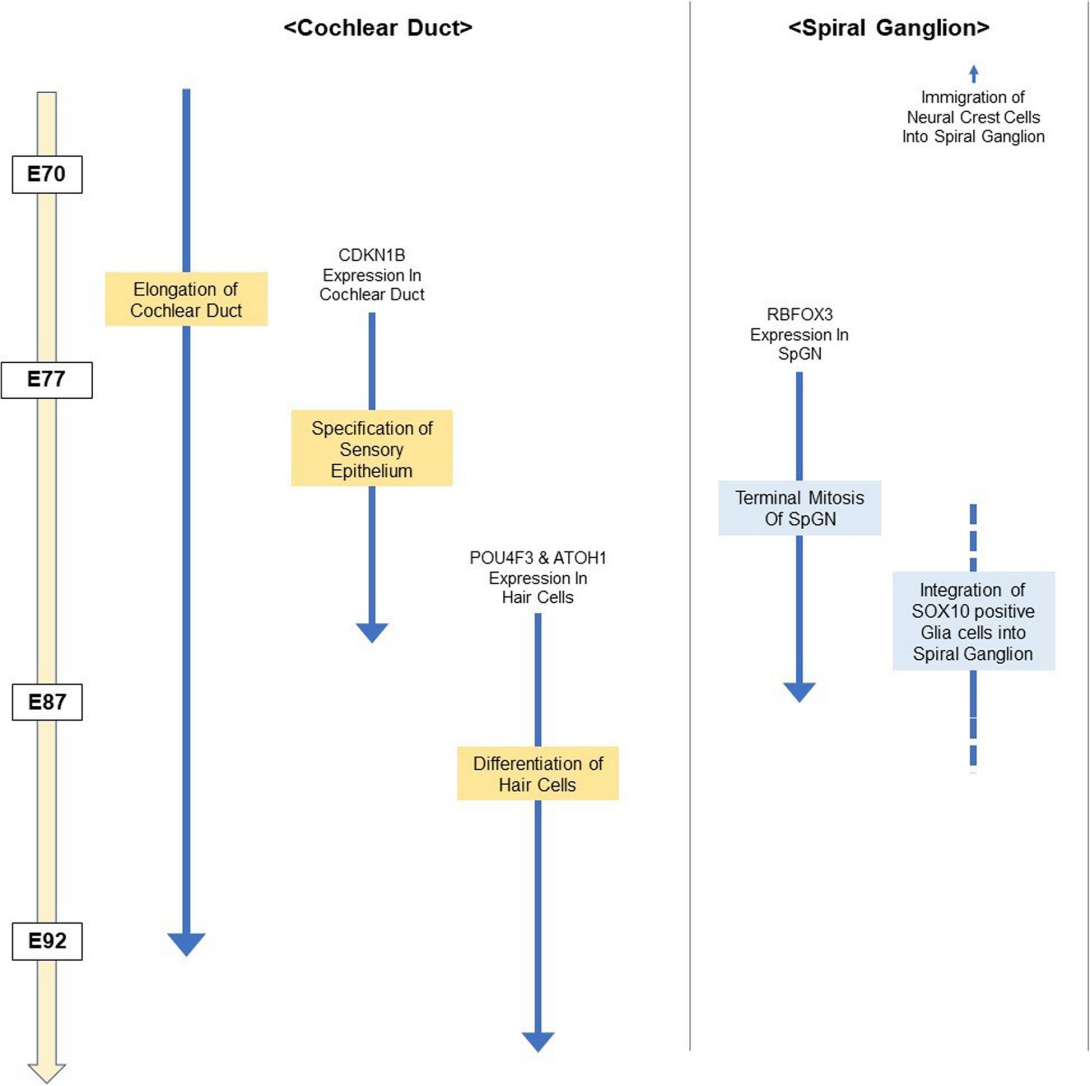
Schematic diagrams showing early cochlear development. The essential developmental steps of early cochlear development in the common marmoset investigated in this study are indicated

The time course of early cochlear development in the common marmoset is summarized in Fig. 14. The essential developmental steps of early cochlear development include elongation of the cochlear duct, specification of the sensory epithelium, and differentiation of hair cells. This schema indicates the temporal relationship between the crucial steps.

**Fig. 15:**
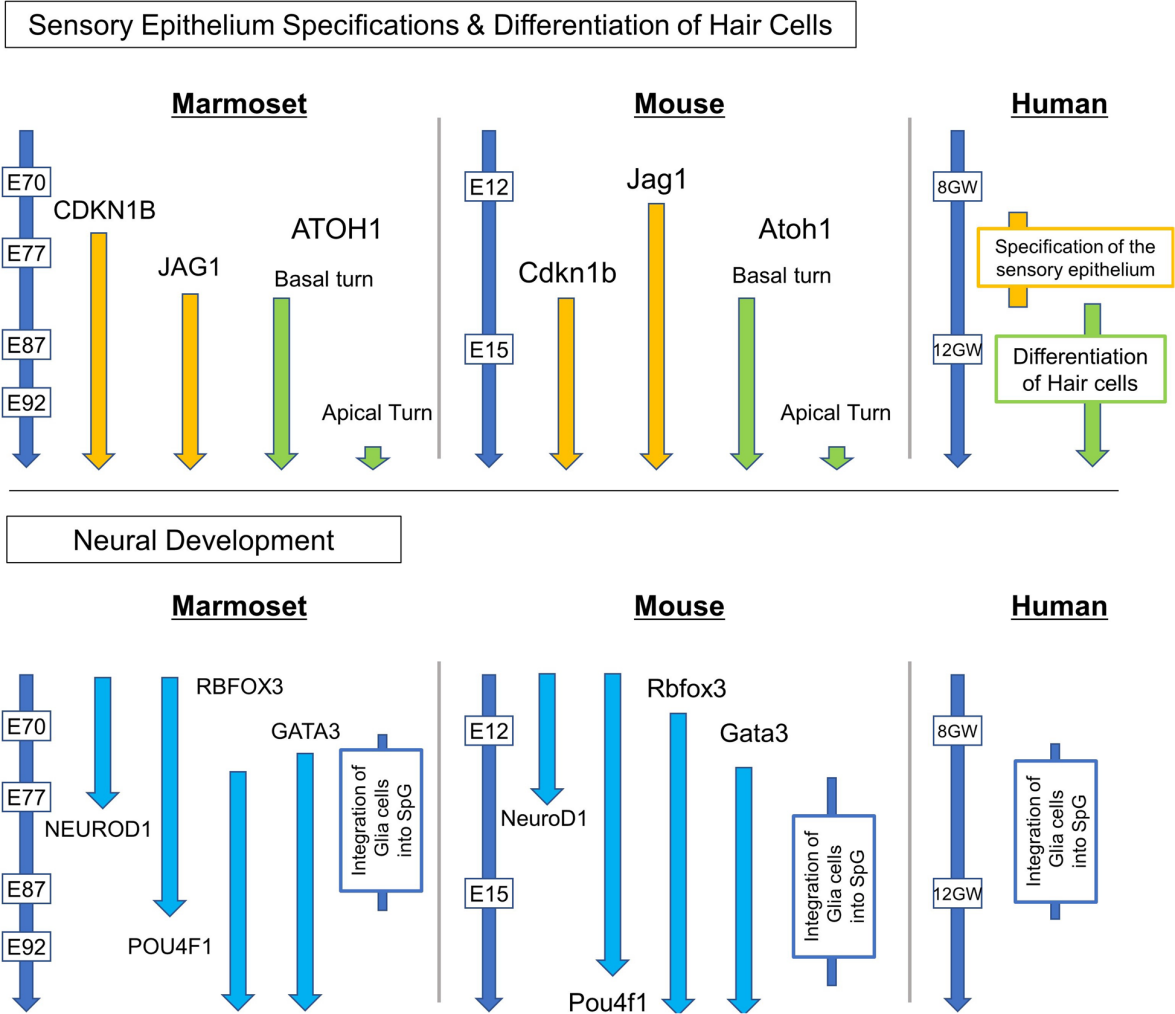
Schematic diagrams showing early cochlear development comparing common marmoset, mouse, and human. This study confirmed that most of the early cochlear developmental steps of the common marmoset were conserved in humans or mice. Gene expression patterns in hair cells development and neural development were well-conserved between the species. However, several inter-species differences were also observed. Expression patterns of the genes associated with the sensory epithelium specifications (CDKN1B and JAG1) showed more significant inter-species differences between the common marmoset and mouse. In marmosets, the progression of differentiation of hair cells from basal to apical turn is about three times slower than in mice

This study confirmed that most of the developmental steps of the common marmoset were conserved in humans or mice. However, several inter-species differences were observed as summarized in the schema (Fig. 15). Two of the most prominent inter-species differences observed in this study were the tempo of the cochlear development, and expression patterns of the genes associated with the sensory epithelium specifications.

So far, although the inter-species differences in the tempos of cochlear development have been underestimated, they may be essential for organogenesis from pluripotent stem cells or organ regeneration [65]. In this study, it was observed that cochlear elongation (from E70 to E92) takes about three weeks, and differentiation of hair cells from basal to apical turn takes about two weeks (from E87 to E101). In contrast, in mice, it has been reported that cochlear elongation took about one week (from E10.75 to E17), [53] and hair cells differentiation needed four days (from E13.5 to E17.5) [19]. This comparison indicates that cochlear development is approximately three times slower in the common marmoset than in mice. Overall, the tempo of cochlear development of the common marmoset was more similar to that of humans than that of mice. In general, the body pattern formation of an embryo, known as segmentation, progresses more slowly in humans than in mice [66, 67]. This process is governed by an oscillating biochemical process known as the segmentation clock [68]. Recently, the inter-species difference in the segmentation clock has been investigated. Reports have shown that this inter-species difference in the tempo of the segmentation clock is caused by the difference in the rates of biochemical reactions; the synthesis and degradation of the critical molecules are two or three times slower in humans than in mice [66]. Our observation indicates that a similar phenomenon that explains the inter-species differences in the tempos of the segmentation clock may also underlie cochlear development.

The inter-species differences in the expression patterns of prosensory domain-specific genes are another prominent feature of this study. The molecular mechanism of prosensory domain specification and subsequent hair cell differentiation is of considerable interest in the field of hearing research, as regeneration of hair cells retracing the developmental steps may be utilized for designing therapy for hearing loss [69, 70]. Our data imply that the procedures used for manipulating rodent cochlear sensory cells cannot be directly used to research primate cells due to the intrinsic inter-species differences in the cell fate determination program. More detailed studies are needed on the species-specific mechanisms that determine sensory cell fate during cochlear development in primates.

## Conclusion

We determined the time course of the essential developmental stages of early cochlear development in the common marmoset, a non-human primate model. In addition, we clarified the similarities and differences in the expression patterns of developmental proteins in the cochlea between previously established rodent models and the primate model. Our data will be helpful for studying early cochlear development in primates and human.

## Data Availability

All data generated or analysed during this study are included in this published article.

## Abbreviations

PRPH: Peripherin
NKCC1: Na-K-Cl cotransporter 1
POU4F3: POU class 4 homeobox 3
ATOH1: Atonal bHLH transcription factor 1
SOX2: SRY-box transcription factor 2
CDKN1B: Cyclin dependent kinase inhibitor 1B
JAG1: Jagged canonical Notch ligand 1
OTX1: Orthodenticle homeobox 1
OTX2: Orthodenticle homeobox 2
PAX2: Paired box 2
GATA3: GATA binding protein 3
ISL1: ISL LIM homeobox 1
PAX8: Paired box 8
DLX5: Distal-less homeobox 5
NEUROD1: Neuronal differentiation 1
POU4F1: POU class 4 homeobox 1
RBFOX3: RNA binding fox-1 homolog 3
MAFB: MAF bZIP transcription factor B
SOX10: SRY-box transcription factor 10
S100B: S100 calcium binding protein B

## References

[1] Stover T, Diensthuber M (2011). Molecular biology of hearing. GMS Curr Top Otorhinolaryngol Head Neck Surg.

[2] Ekdale EG (2016). Form and function of the mammalian inner ear. J Anat.

[3] Roccio M, Senn P, Heller S (2020). Novel insights into inner ear development and regeneration for targeted hearing loss therapies. Hear Res.

[4] Wu DK, Kelley MW (2012). Molecular mechanisms of inner ear development. Cold Spring Harb Perspect Biol.

[5] Locher  H, Frijns JH, van Iperen L, de Groot JC, Huisman MA, Chuva de Sousa Lopes SM (2013). Neurosensory development and cell fate determination in the human cochlea. Neural Dev.

[6] Ota CY, Kimura RS (1980). Ultrastructural study of the human spiral ganglion. Acta Otolaryngol.

[7] Hosoya M, Fujioka M, Murayama AY, Okano H, Ogawa K (2021). The common marmoset as suitable nonhuman alternative for the analysis of primate cochlear development. FEBS J.

[8] Hosoya M, Fujioka M, Murayama AY, Ozawa H, Okano H, Ogawa K (2021). Neuronal development in the cochlea of a nonhuman primate model, the common marmoset. Dev Neurobiol.

[9] Hosoya M, Fujioka M, Murayama AY, Ogawa K, Okano H, Ozawa H (2021). Dynamic Spatiotemporal Expression Changes in Connexins of the Developing Primate's Cochlea. Genes..

[10] Hosoya M, Fujioka M, Murayama AY, Ogawa K, Okano H, Ozawa H (2021). Dynamic Spatiotemporal Expression Changes in Connexins of the Developing Primate’s Cochlea. Genes.

[11] Matsuzaki S, Hosoya M, Okano H, Fujioka M, Ogawa K (2018). Expression pattern of EYA4 in the common marmoset (Callithrix jacchus) cochlea. Neurosci Lett.

[12] Hosoya M, Fujioka M, Okano H, Ogawa K (2016). Distinct Expression Pattern of a Deafness Gene, KIAA1199, in a Primate Cochlea. Biomed Res Int.

[13] Suzuki N, Hosoya M, Oishi N, Okano H, Fujioka M, Ogawa K (2016). Expression pattern of wolframin, the WFS1 (Wolfram syndrome-1 gene) product, in common marmoset (Callithrix jacchus) cochlea. NeuroReport.

[14] Hosoya M, Fujioka M, Ogawa K, Okano H (2016). Distinct Expression Patterns Of Causative Genes Responsible For Hereditary Progressive Hearing Loss In Non-Human Primate Cochlea. Sci Rep.

[15] Sun Z, Cheng Z, Gong N, Xu Z, Jin C, Wu H (2021). Neural presbycusis at ultra-high frequency in aged common marmosets and rhesus monkeys. Aging (Albany NY).

[16] Okano H (2021). Current Status of and Perspectives on the Application of Marmosets in Neurobiology. Annu Rev Neurosci.

[17] Hearn JP, Lunn SF, Burden FJ, Pilcher MM (1975). Management of marmosets for biomedical research. Lab Anim.

[18] Xiang M, Gan L, Li D, Chen ZY, Zhou L, O'Malley BW (1997). Essential role of POU-domain factor Brn-3c in auditory and vestibular hair cell development. Proc Natl Acad Sci U S A.

[19] Mulvaney J, Dabdoub A (2012). Atoh1, an essential transcription factor in neurogenesis and intestinal and inner ear development: function, regulation, and context dependency. J Assoc Res Otolaryngol.

[20] Bermingham NA, Hassan BA, Price SD, Vollrath MA, Ben-Arie N, Eatock RA (1999). Math1: an essential gene for the generation of inner ear hair cells. Science.

[21] Kiernan AE, Pelling AL, Leung KK, Tang AS, Bell DM, Tease C (2005). Sox2 is required for sensory organ development in the mammalian inner ear. Nature.

[22] Dabdoub A, Puligilla C, Jones JM, Fritzsch B, Cheah KS, Pevny LH (2008). Sox2 signaling in prosensory domain specification and subsequent hair cell differentiation in the developing cochlea. Proc Natl Acad Sci U S A.

[23] Chen P, Segil N (1999). p27(Kip1) links cell proliferation to morphogenesis in the developing organ of Corti. Development.

[24] Morrison A, Hodgetts C, Gossler A, Hrabe de Angelis M, Lewis J (1999). Expression of Delta1 and Serrate1 (Jagged1) in the mouse inner ear. Mech Dev.

[25] Kiernan AE, Xu J, Gridley T (2006). The Notch ligand JAG1 is required for sensory progenitor development in the mammalian inner ear. PLoS Genet.

[26] Murata J, Tokunaga A, Okano H, Kubo T (2006). Mapping of notch activation during cochlear development in mice: implications for determination of prosensory domain and cell fate diversification. J Comp Neurol.

[27] Murata J, Ohtsuka T, Tokunaga A, Nishiike S, Inohara H, Okano H (2009). Notch-Hes1 pathway contributes to the cochlear prosensory formation potentially through the transcriptional down-regulation of p27Kip1. J Neurosci Res.

[28] Bok J, Chang W, Wu DK (2007). Patterning and morphogenesis of the vertebrate inner ear. Int J Dev Biol.

[29] Chatterjee S, Kraus P, Lufkin T (2010). A symphony of inner ear developmental control genes. BMC Genet.

[30] Brigande JV, Kiernan AE, Gao X, Iten LE, Fekete DM (2000). Molecular genetics of pattern formation in the inner ear: do compartment boundaries play a role?. Proc Natl Acad Sci U S A.

[31] Morsli H, Tuorto F, Choo D, Postiglione MP, Simeone A, Wu DK (1999). Otx1 and Otx2 activities are required for the normal development of the mouse inner ear. Development.

[32] Burton Q, Cole LK, Mulheisen M, Chang W, Wu DK (2004). The role of Pax2 in mouse inner ear development. Dev Biol.

[33] van der Wees J, van Looij MA, de Ruiter MM, Elias H, van der Burg H, Liem SS (2004). Hearing loss following Gata3 haploinsufficiency is caused by cochlear disorder. Neurobiol Dis.

[34] Luo XJ, Deng M, Xie X, Huang L, Wang H, Jiang L (2013). GATA3 controls the specification of prosensory domain and neuronal survival in the mouse cochlea. Hum Mol Genet.

[35] Huang M, Sage C, Li H, Xiang M, Heller S, Chen ZY (2008). Diverse expression patterns of LIM-homeodomain transcription factors (LIM-HDs) in mammalian inner ear development. Dev Dyn.

[36] Radde-Gallwitz K, Pan L, Gan L, Lin X, Segil N, Chen P (2004). Expression of Islet1 marks the sensory and neuronal lineages in the mammalian inner ear. J Comp Neurol.

[37] He D, Guo R, Zheng D, Xu M, Li P, Guo L (2020). Transcription factor Isl1 is dispensable for the development of the mouse prosensory region. Cytotechnology.

[38] Kim WY, Fritzsch B, Serls A, Bakel LA, Huang EJ, Reichardt LF (2001). NeuroD-null mice are deaf due to a severe loss of the inner ear sensory neurons during development. Development.

[39] Liu M, Pereira FA, Price SD, Chu MJ, Shope C, Himes D (2000). Essential role of BETA2/NeuroD1 in development of the vestibular and auditory systems. Genes Dev.

[40] Lawoko-Kerali G, Rivolta MN, Lawlor P, Cacciabue-Rivolta DI, Langton-Hewer C, van Doorninck JH (2004). GATA3 and NeuroD distinguish auditory and vestibular neurons during development of the mammalian inner ear. Mech Dev.

[41] Huang EJ, Liu W, Fritzsch B, Bianchi LM, Reichardt LF, Xiang MQ (2001). Brn3a is a transcriptional regulator of soma size, target field innervation and axon pathfinding of inner ear sensory neurons. Development.

[42] Deng M, Yang H, Xie X, Liang G, Gan L (2014). Comparative expression analysis of POU4F1, POU4F2 and ISL1 in developing mouse cochleovestibular ganglion neurons. Gene Expr Patterns.

[43] Lawoko-Kerali G, Rivolta MN, Holley M (2002). Expression of the transcription factors GATA3 and Pax2 during development of the mammalian inner ear. J Comp Neurol.

[44] Yu WM, Appler JM, Kim YH, Nishitani AM, Holt JR, Goodrich LV (2013). A Gata3-Mafb transcriptional network directs post-synaptic differentiation in synapses specialized for hearing. Elife.

[45] D'Amico-Martel A, Noden DM (1983). Contributions of placodal and neural crest cells to avian cranial peripheral ganglia. American Journal of Anatomy.

[46] Sandell LL, Butler Tjaden NE, Barlow AJ, Trainor PA (2014). Cochleovestibular nerve development is integrated with migratory neural crest cells. Dev Biol.

[47] Locher H, de Groot JC, van Iperen L, Huisman MA, Frijns JH, Chuva de Sousa Lopes SM (2014). Distribution and development of peripheral glial cells in the human fetal cochlea. PLoS One.

[48] Streeter GL (1906). On the development of the membranous labyrinth and the acoustic and facial nerves in the human embryo. American Journal of Anatomy.

[49] Lim R, Brichta AM (2016). Anatomical and physiological development of the human inner ear. Hear Res.

[50] Pujol R, Lavigne-Rebillard M (1985). Early stages of innervation and sensory cell differentiation in the human fetal organ of Corti. Acta Otolaryngol Suppl.

[51] Lavigne-Rebillard M, Pujol R (1987). Surface aspects of the developing human organ of Corti. Acta Otolaryngol Suppl.

[52] Hall JW (2000). Development of the ear and hearing. J Perinatol.

[53] Cantos R, Cole LK, Acampora D, Simeone A, Wu DK (2000). Patterning of the mammalian cochlea. Proc Natl Acad Sci U S A.

[54] Kelley MW (2006). Regulation of cell fate in the sensory epithelia of the inner ear. Nat Rev Neurosci.

[55] Brooker R, Hozumi K, Lewis J (2006). Notch ligands with contrasting functions: Jagged1 and Delta1 in the mouse inner ear. Development.

[56] Vendrell V, Lopez-Hernandez I, Duran Alonso MB, Feijoo-Redondo A, Abello G, Galvez H (2015). Otx2 is a target of N-myc and acts as a suppressor of sensory development in the mammalian cochlea. Development.

[57] Pechriggl EJ, Bitsche M, Glueckert R, Rask-Andersen H, Blumer MJ, Schrott-Fischer A (2015). Development of the innervation of the human inner ear. Dev Neurobiol.

[58] Bouchard M, de Caprona D, Busslinger M, Xu P, Fritzsch B (2010). Pax2 and Pax8 cooperate in mouse inner ear morphogenesis and innervation. BMC Dev Biol.

[59] Acampora D, Merlo GR, Paleari L, Zerega B, Postiglione MP, Mantero S (1999). Craniofacial, vestibular and bone defects in mice lacking the Distal-less-related gene Dlx5. Development.

[60] Robledo RF, Lufkin T (2006). Dlx5 and Dlx6 homeobox genes are required for specification of the mammalian vestibular apparatus. Genesis.

[61] Hafidi A, Despres G, Romand R (1993). Ontogenesis of type II spiral ganglion neurons during development: peripherin immunohistochemistry. Int J Dev Neurosci.

[62] Kim KK, Adelstein RS, Kawamoto S (2009). Identification of Neuronal Nuclei (NeuN) as Fox-3, a New Member of the Fox-1 Gene Family of Splicing Factors. J Biol Chem.

[63] Mullen RJ, Buck CR, Smith AM (1992). NeuN, a neuronal specific nuclear protein in vertebrates. Development.

[64] Nishimura K, Noda T, Dabdoub A (2017). Dynamic Expression of Sox2, Gata3, and Prox1 during Primary Auditory Neuron Development in the Mammalian Cochlea. PLoS ONE.

[65] van den Ameele J, Tiberi L, Vanderhaeghen P, Espuny-Camacho I (2014). Thinking out of the dish: what to learn about cortical development using pluripotent stem cells. Trends Neurosci.

[66] Matsuda M, Hayashi H, Garcia-Ojalvo J, Yoshioka-Kobayashi K, Kageyama R, Yamanaka Y (2020). Species-specific segmentation clock periods are due to differential biochemical reaction speeds. Science.

[67] Ebisuya M, Briscoe J (2018). What does time mean in development?. Development.

[68] Oates AC, Morelli LG, Ares S (2012). Patterning embryos with oscillations: structure, function and dynamics of the vertebrate segmentation clock. Development.

[69] Mizutari K, Fujioka M, Hosoya M, Bramhall N, Okano HJ, Okano H (2013). Notch inhibition induces cochlear hair cell regeneration and recovery of hearing after acoustic trauma. Neuron.

[70] McLean WJ, Yin X, Lu L, Lenz DR, McLean D, Langer R (2017). Clonal Expansion of Lgr5-Positive Cells from Mammalian Cochlea and High-Purity Generation of Sensory Hair Cells. Cell Rep.

